# IFITM proteins assist cellular uptake of diverse linked chemotypes

**DOI:** 10.1126/science.abl5829

**Published:** 2022-12-08

**Authors:** Kevin Lou, Douglas R. Wassarman, Tangpo Yang, YiTing Paung, Ziyang Zhang, Thomas A. O’Loughlin, Megan K. Moore, Regina K. Egan, Patricia Greninger, Cyril H. Benes, Markus A. Seeliger, Jack Taunton, Luke A. Gilbert, Kevan M. Shokat

**Affiliations:** 1 Department of Cellular and Molecular Pharmacology, University of California, San Francisco, San Francisco, CA 94158, United States.; 2 Howard Hughes Medical Institute, University of California, San Francisco, San Francisco, CA 94158, United States.; 3 Department of Pharmacological Sciences, Stony Brook University, Stony Brook, New York 11794-8651, United States.; 4 Department of Chemistry, University of California, Berkeley, Berkeley, 94720, CA, United States.; 5 Helen Diller Family Comprehensive Cancer Center, University of California, San Francisco, San Francisco, CA 94158, United States.; 6 Department of Urology, University of California, San Francisco, San Francisco, CA 94158, United States.; 7 Center for Cancer Research, Massachusetts General Hospital Cancer Center, Charlestown, MA 02129, United States.; 8 Department of Medicine, Harvard Medical School, Boston, MA 02115, United States.; 9 Innovative Genomics Institute, University of California, San Francisco, San Francisco, CA 94158, United States.; 10 Arc Institute, Palo Alto, CA, 94304, United States.

## Abstract

The search for cell permeable drugs has conventionally focused on low molecular weight, non-polar, and rigid chemical structures. However, emerging therapeutic strategies break traditional drug design rules by employing flexibly linked chemical entities composed of more than one ligand. Using complementary genome-scale chemical-genetic approaches we identified an endogenous chemical uptake pathway involving interferon induced transmembrane proteins (IFITMs) that modulates the cell permeability of a prototypical biopic inhibitor of MTOR (RapaLink-1, MW: 1784 g/mol). We devised additional linked inhibitors targeting BCR-ABL1 (DasatiLink-1, MW: 1518 g/mol) and EIF4A1 (BisRoc-1, MW: 1466 g/mol) whose uptake was facilitated by IFITMs. We also found that IFITMs moderately assisted some proteolysis targeting chimeras (PROTACs) and examined the physicochemical requirements for involvement of this uptake pathway.

## 1 sentence summary

Chemical-genetic approaches elucidate uptake pathway for molecules breaking traditional drug design rules.

Any therapeutic molecule that binds to an intracellular target must first cross the cell membrane. Retrospective analyses of compound libraries and their biological activities have yielded empirical guidelines (e.g. Lipinski’s rule of five) that enrich for lead-like scaffolds with high passive permeability and largely define modern drug-like chemical space (1–3). While these principles have been useful for streamlining the search for new therapeutics, many important intracellular drug targets are currently refractory to inhibition by these compact, hydrophobic, and rigid molecules. An emerging design framework that seeks to address these challenges involves increasing pharmacological complexity by linking multiple ligands into a single chemical entity (a linked chemotype). Linked chemotypes can have enhanced potency, greater selectivity, and the capacity to induce the association of more than one target (4–11). This modular rapid access to high molecular weight, amphiphilicity, and rotational flexibility can provide useful chemical probes and therapeutic leads for intracellular targets, as long as the resulting molecules remain cell permeable.

Mechanisms to understand and predict the cell permeability of linked chemotypes, however, remain limited. Other medium-to-high molecular weight therapeutics such as natural products and synthetic macrocycles often comprise highly tailored arrangements of polar/non-polar functionality that allow switching between membrane-favored and aqueous-favored conformations to enable passive permeability through membranes (12). Additionally, cell penetrating proteins/peptides commonly require appendage of highly charged moieties to enable electrostatic interactions with the plasma membrane and subsequent internalization (13–15). Studies involving the most rapidly expanding linked chemotype class, proteolysis targeting chimeras (PROTACs) (16), provide varying insights into the determinants of cell permeability (17–22). Despite their atypical properties, PROTACs and additional large molecules such as the dimeric immunophilin ligand rimiducid have shown in-cell activity robust enough to enter clinical trials (16, 23).

**Fig. 1.**
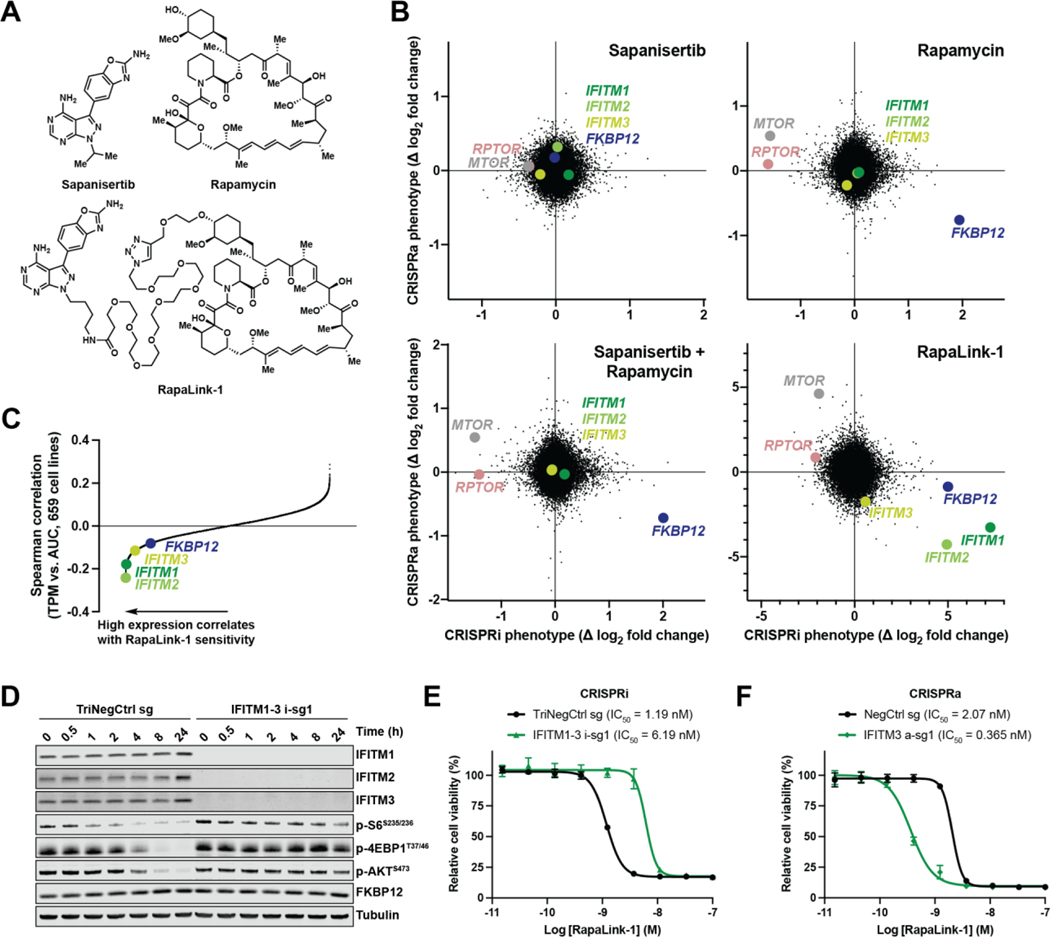
IFITMs promote the cellular activity of a bitopic MTOR inhibitor. (**A**) Chemical structures of MTOR inhibitors. (**B**) Gene phenotypes from genome-scale CRISPRi and CRISPRa screens in K562 cells. Genes involved in MTOR complex 1 (*MTOR* and *RPTOR*), a requisite rapamycin inhibitory complex partner (*FKBP12*), and clade I IFITMs (*IFITM1*, *IFITM2*, and *IFITM3*) are highlighted. Data represent two biological replicates. (**C**) Spearman correlation coefficients between RapaLink-1 sensitivity, as measured by dose-response data, and transcript abundance, as measured by RNA sequencing (see also fig. S4). Dose-response data are expressed as area under the curve (AUC) and RNA sequencing data are expressed as transcripts per million (TPM). Genes are highlighted as in (B). (**D**) Immunoblots of K562 CRISPRi cells expressing sgRNAs treated with RapaLink-1 (3 nM) for the times indicated. (**E** and **F**) Viability of K562 CRISPRi (E) or CRISPRa (F) cells expressing sgRNAs treated with RapaLink-1. Data represent means of three biological replicates; error bars denote SD.

Given this discrepancy between the favorable biological activity of many large, bivalent molecules and traditional concepts of passive permeability, we inferred that linked chemotypes might hijack cellular processes to assist with passage through the cell membrane. We selected as an example a bitopic inhibitor of MTOR, RapaLink-1 (7), whose molecular weight (1784 g/mol) falls well beyond common guidelines (≤ 500 g/mol) (1), and even beyond that of typical PROTACs (800–1200 g/mol) (table S1) (18). RapaLink-1’s atypical structure, composed of the allosteric MTOR inhibitor rapamycin and the active-site inhibitor sapanisertib linked by an 8-unit polyethylene glycol (PEG8) tether (Fig. 1A), confers enhanced selectivity for MTOR complex 1 over MTOR complex 2 (7, 24, 25). The molecule is highly active *in vivo*, penetrates the blood-brain barrier, and serves as a prototype for the clinical candidate RMC-5552 (7, 24–27), establishing itself as a drug-like compound that defies most traditional notions of drug-like structure. We hypothesized that cellular mechanisms assisting RapaLink-1’s cytoplasmic entry could be identified by systematically perturbing genes that modulate the molecule’s ability to reach and inhibit its intracellular target.

## Complementary genome-scale chemical-genetic approaches identify IFITMs as regulators of RapaLink-1 cellular activity

We probed canonical protein coding genes for cellular factors that determine RapaLink-1 uptake and sensitivity using a dCas9-based CRISPRi/a functional genomics platform (28, 29). Gene expression inhibition and activation, through CRISPRi and CRISPRa respectively, act as complementary approaches to map chemical-genetic interactions at genome-scale. In particular, genes displaying strong mirrored phenotypes (resistance upon knockdown and sensitivity upon overexpression) are likely to be directly involved in a small molecule’s mechanism of action. This integrated approach to identifying physiologically relevant chemical-genetic interactions was proposed by Jost et al. and its utility has recently been reviewed (30, 31). In addition to the bitopic inhibitor, we also assessed sapanisertib, rapamycin, and an unlinked control (a 1:1 mixture of sapanisertib and rapamycin) to distinguish chemical-genetic interactions specific to the linked chemotype (Fig. 1A).

Patient-derived chronic myeloid leukemia (CML) cells, K562, pre-engineered to express CRISPRi or CRISPRa machinery, were transduced with their respective genome-scale sgRNA libraries, selected with puromycin to remove non-transduced cells, and treated with DMSO, sapanisertib, rapamycin, sapanisertib + rapamycin, or RapaLink-1. The experiments were conducted with high replicate reproducibility (fig. S1, A to D), and data from the genome-scale CRISPRi (data file S1 and data file S2) and CRISPRa (data file S3 and data file S4) screens were juxtaposed to highlight genes that displayed mirrored phenotypes (Fig. 1B). This arrangement distributes genes which functionally synergize with the inhibitor in the lower right (e.g. *FKBP12*, the required inhibitory complex partner of rapamycin) and those which antagonize the inhibitor in the upper left (e.g. *MTOR*, the direct target) (30, 31). Chemical-genetic interactions with MTOR signaling components, particularly the Ragulator complex (*RRAGA*, *RRAGC*, and *LAMTOR1–5*) and nodes downstream of PI3K/AKT (*TSC1*, *TSC2*, and *RHEB*), were observed across multiple inhibitor conditions (fig. S2, A and B), consistent with known pathway relationships (32) and prior functional genomics studies (33, 34).

A distinct set of chemical-genetic interactions were identified as top hits with RapaLink-1 and not with any of the non-linked molecules tested, suggesting the involvement of a biological pathway that promotes the activity of the linked chemotype. The expression of members of a highly homologous gene family, interferon induced transmembrane proteins (IFITMs) *IFITM1*, *IFITM2*, and *IFITM3* (35), synergized with the activity of RapaLink-1 and not its non-linked counterparts, sapanisertib and rapamycin (Fig. 1B). To validate this finding, we tested sgRNAs targeting *IFITM1–3* individually for transcriptional repression or activation (fig. S3A and table S2). CRISPRi-mediated knockdown of *IFITM1–3* was potent and selective (fig. S3B). CRISPRa-mediated overexpression was also potent although we observed variable cross activation between family members (fig. S3C), possibly due to concerted transcriptional regulation of these genes, which are adjacent to each other on chromosome 11 (fig. S3A) (36). We individually confirmed that top screen hits, including *FKBP12* and *IFITM1–3*, synergized with RapaLink-1 in a competitive growth assay and also validated that the *IFITM1–3* chemical-genetic interaction was specific to the linked chemotype (fig. S3, D and E).

Seeking to generalize these observations beyond a single cell type, we employed an independent chemical-genetic approach correlating MTOR inhibitor sensitivity data with basal gene expression in diverse *in vitro* models (37–39). Over 500 cancer cell lines were assessed for sensitivity to sapanisertib, rapamycin, or RapaLink-1 (measured by area under the dose-response curve). These measurements were correlated with gene transcript abundance (measured by RNA sequencing) across the cell lines to identify predictive biomarkers for compound sensitivity or resistance. High expression of any of the three IFITM family members was strongly associated with enhanced RapaLink-1 sensitivity across 659 cell lines, and *IFITM2* was notably the single most associated sensitizing biomarker (negative correlation) for Rapalink-1 (Fig. 1C and data file S5). This correlation was absent for sapanisertib and rapamycin (fig. S4, A to C, and data file S5), recapitulating the CRISPRi/a screens. Together, our analysis of the CRISPRi/a screens and large-scale chemogenomic cell line profiling experiments suggested a role of IFITMs in promoting the activity of RapaLink-1 across diverse cell types and levels of IFITM expression.

## IFITMs promote RapaLink-1 pharmacodynamic target engagement

To unmask potentially overlapping IFITM functions, we knocked down *IFITM1*, *IFITM2*, and *IFITM3* expression simultaneously by co-expressing three different targeting sgRNAs (table S2) (40). While multigene knockdown was potent (fig. S5A), we did not observe baseline changes in cell viability (fig. S5B). *IFITM1–3* triple knockdown ablated MTOR inhibition by 3 nM RapaLink-1 in cells, as determined by intracellular markers of MTOR pathway signaling, phospho-S6^S235/236^, phospho-4EBP1^T37/46^, and phospho-AKT^S473^ (Fig. 1D), and conferred resistance to the linked molecule (Fig. 1E and fig. S5C). Overall, IFITM expression perturbation by CRISPRi and CRISPRa caused a combined 29.5-fold modulation in cellular potency of the molecule (Fig. 1, E and F). As has been observed previously (7), RapaLink-1 requires multiple hours to achieve maximal pharmacodynamic inhibition (Fig. 1D). This contrasts with the typical finding that small molecules reach their intracellular targets on the seconds-to-minutes timescale (41) and may reflect how linked chemotypes exhibit distinct permeability characteristics from traditional drug-like molecules.

Neither of the non-linked MTOR inhibitors tested demonstrated chemical-genetic interactions with *IFITM1–3*, thus IFITMs likely do not directly modulate MTOR signaling, but instead cooperate with some aspect of RapaLink-1 not shared with the other inhibitors. Clade I IFITM family members, *IFITM1–3*, are closely related broad spectrum viral restriction factors that localize to the plasma and endolysosomal membranes (42–45). They are thought to perform their antiviral function, in part, by rendering local membrane characteristics at the viral-endosomal juncture unfavorable for viral entry (46), although in some cases viruses can also hijack IFITMs to facilitate entry and infection (47). In addition to their established immunologic function, clade I IFITMs are also reported to modulate an oncogenic phenotype (48), affect placenta formation (49), and contribute to cellular homeostasis (44). In turn, Rapalink-1 might interact with IFITMs through a cellular pathway that promotes the uptake of the large molecule.

## A fluorescent RapaLink-1 analog reveals a role for IFITMs in linked chemotype uptake

**Fig. 2.**
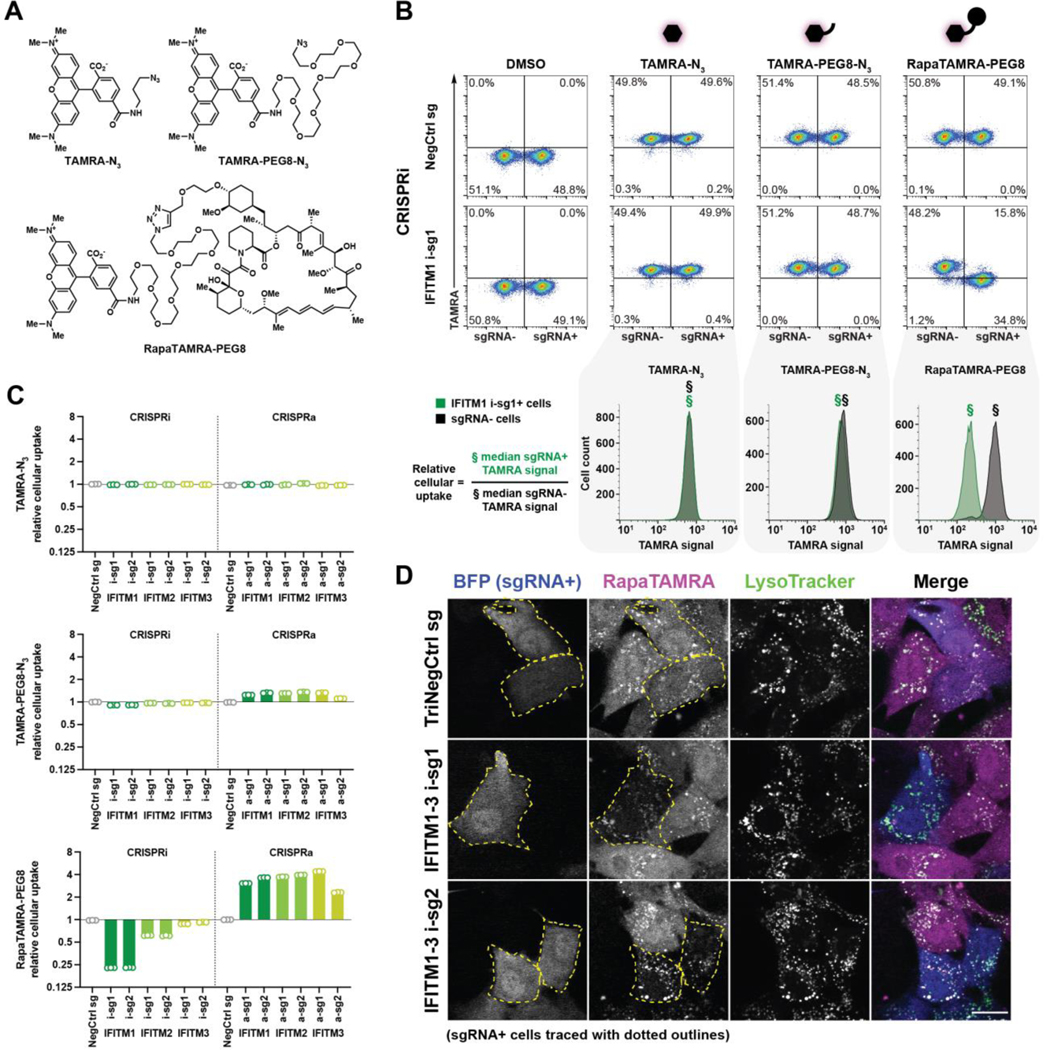
IFITMs promote the cellular uptake of linked chemotypes. (**A**) Chemical structures of fluorescent RapaLink-1 analogs. (**B**) Measurement of fluorescent molecule uptake in K562 CRISPRi cells expressing sgRNAs (sgRNA+). Cells were incubated with TAMRA-N_3_ (10 nM), TAMRA-PEG8-N_3_ (1 μM), or RapaTAMRA-PEG8 (1 nM) for 24 h. Uptake modulation by sgRNAs was quantified by internal normalization to non-transduced cells (sgRNA-) present within the mixture (i.e. relative cellular uptake). Data representative of three biological replicates. (**C**) Changes in uptake of fluorescent molecules by sgRNAs targeting *IFITM1–3* as in (B and fig. S6A). Relative cellular uptake < 1 indicates decreased uptake and > 1 indicates increased uptake. Data represent means of three biological replicates. (**D**) Confocal microscopy images of RPE-1 CRISPRi cells expressing indicated sgRNAs (blue) and treated for 24 h with RapaTAMRA-PEG8 (magenta) and LysoTracker (green). sgRNA+ cells are traced with dotted outlines (yellow) in left two columns for clarity. Scale bar denotes 20 μm.

To explore our uptake hypothesis, we created a fluorescent analog of RapaLink-1 to directly observe the effect of IFITM expression on accumulation of the linked chemotype in live cells. This fluorescent molecule, RapaTAMRA-PEG8, was designed by replacing the adenosine triphosphate (ATP)-site binding element in RapaLink-1 with tetramethylrhodamine (TAMRA), resulting in a fluorescent derivative that closely mimics the physicochemical properties of the original molecule (Fig. 2A and table S1) (27). Analogs representing partial components of RapaTAMRA-PEG8, TAMRA-N_3_ and TAMRA-PEG8-N_3_, were additionally evaluated to assess whether the uptake pathway extended to generic compact-hydrophobic or linked-amphiphilic chemotypes respectively (Fig. 2A). We quantified accumulation of these molecules by flow cytometry using a quantitative live cell fluorescence uptake assay in which a mixture of transduced (sgRNA+) and non-transduced (sgRNA-) cells were equally exposed to compound within the same well (Fig. 2B and fig. S6A). Changes in cellular uptake resulting from CRISPRi/a expression modulation by sgRNAs (fig. S3, A to C) again revealed a chemotype-specific IFITM dependency pattern (Fig. 2, B and C, and fig. S6A). Both linked chemotypes, TAMRA-PEG8-N_3_ and RapaTAMRA-PEG8, demonstrated decreased uptake upon knockdown of *IFITM1–3* and increased uptake upon overexpression. The linker-less chemotype, TAMRA-N_3_, in contrast exhibited no such chemical-genetic interactions. CRISPRi/a-induced uptake differences observed for RapaTAMRA-PEG8 correlated strongly with resistance and sensitivity phenotypes for RapaLink-1 (fig. S6B), suggesting a direct association between measured uptake and functional target inhibition. The observation that uptake of TAMRA-PEG8-N_3_, a generic linked chemotype not specifically bound by any cellular protein, was also IFITM-assisted suggested that this uptake mechanism might also be used by other linked molecules.

Additionally, we assessed the role of IFITMs on the subcellular localization of RapaTAMRA-PEG8 by confocal microscopy in a human non-transformed cell line, RPE-1, pre-engineered to express CRISPRi machinery. Again a mixture of sgRNA+ and sgRNA- cells were imaged in the same well following equal exposure to fluorescent compound. *IFITM1–3* triple-knockdown significantly reduced the amount of RapaTAMRA-PEG8 entering the intracellular compartment (Fig. 2D and fig. S6C). A reduction in signal was also observed within the endolysosomal compartment (Fig. 2D and fig. S6C), suggesting that IFITMs, which localize to the plasma membrane as well as endolysosomal membranes (43–45), may play a role in facilitating RapaTAMRA-PEG8 uptake through endocytic vesicles and into the intracellular space. Consistent with this, our functional genomics screens identified RapaLink-1-specific chemical-genetic interactions among endosomal (*ARF6*, *VPS26A*, *VPS29*, and *VPS35*) and sterol (*OSBP*, *GRAMD1A*, *INSIG1*, and *SCAP*) regulatory genes (fig. S7, A and B). This, in part, resembles IFITMs’ roles as antiviral effectors in which biophysical interactions with incoming viral particles (50) and membrane sterols (51) may hinder or assist infection of target cells (43–45). Considering the large diversity of viruses IFITMs are described to interact with, we hypothesized that the uptake assistance afforded to RapaLink-1 and RapaTAMRA-PEG8 by IFITMs might also extend to other linked chemotypes with similar physicochemical properties.

## DasatiLink-1 is an IFITM-assisted bitopic inhibitor of BCR-ABL1 with enhanced selectivity

**Fig. 3.**
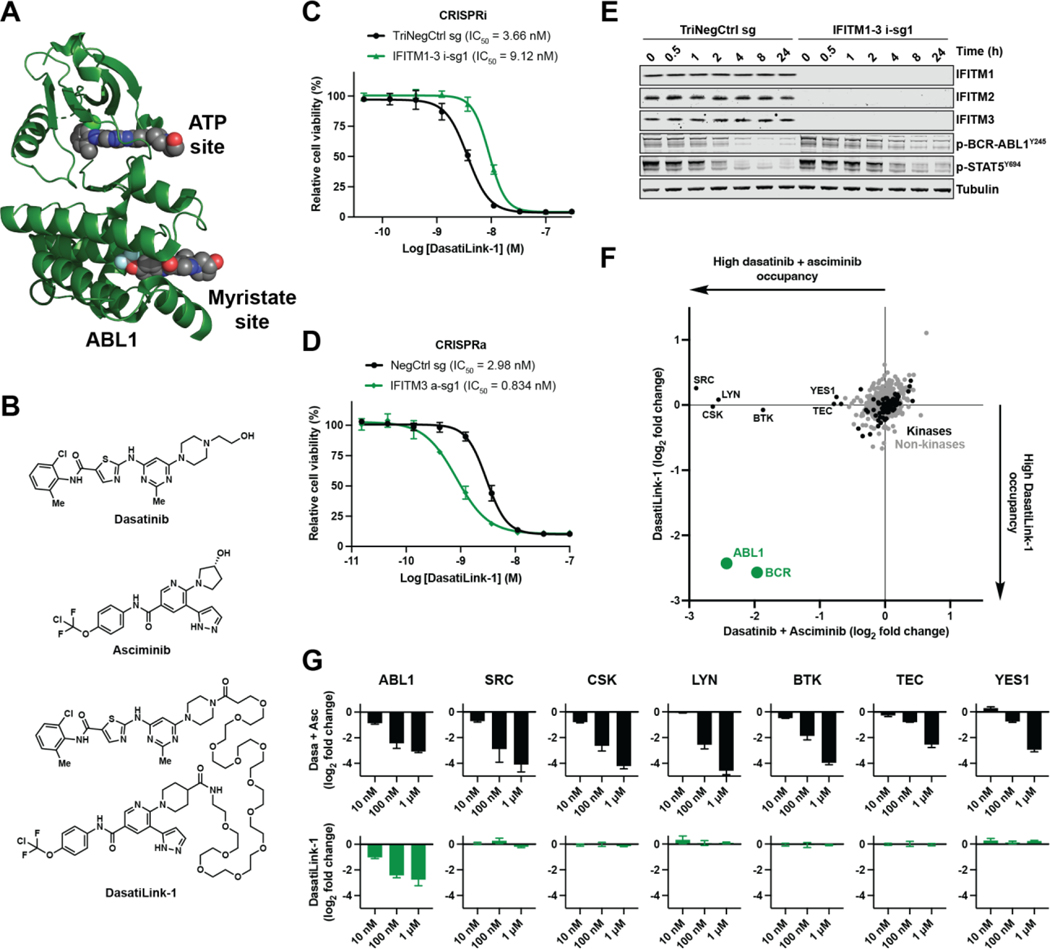
Design and characterization of an IFITM-assisted bitopic BCR-ABL1 inhibitor. (**A**) Molecular model of ABL1 kinase domain. The model was constructed by aligning two crystal structures: one bound to dasatinib (PDB, 2GQG) and one bound to asciminib (PDB, 5MO4). (**B**) Chemical structures of BCR-ABL1 inhibitors. (**C** and **D**) Viability of K562 CRISPRi (C) or CRISPRa (D) cells expressing sgRNAs treated with DasatiLink-1. Data represent means of three biological replicates; error bars denote SD. (**E**) Immunoblots of K562 CRISPRi cells expressing sgRNAs treated with DasatiLink-1 (5 nM) for the times indicated (**F**) In-cell kinase occupancy profiling of DasatiLink-1 and an unlinked control (a 1:1 mixture of dasatinib and asciminib) at equimolar concentration (100 nM). Data represent means of three biological replicates. (**G**) As in (F) for kinases occupied following 10 nM, 100 nM, and 1 μM inhibitor treatments; error bars denote SD.

To explore the generalizability of this IFITM-promoted cellular uptake mechanism, we designed, synthesized, and characterized a bitopic inhibitor that is, aside from being a linked molecule, compositionally unrelated to RapaLink-1. This inhibitor targets a different intracellular protein, BCR-ABL1, a fusion oncoprotein associated with CML and other leukemias (52). BCR-ABL1 harbors two well-defined small molecule binding sites within its kinase domain (Fig. 3A): the ATP pocket (53), which is targeted by five clinically approved compounds (e.g. dasatinib) (54), and the myristoyl pocket (55), which is targeted by the clinical inhibitor asciminib (56). These sites can also be bound by the two classes of inhibitors simultaneously (55–57). The two pockets span a similar distance as those engaged by RapaLink-1 in MTOR (7), suggesting that a similar bitopic inhibitor linkage strategy could apply to BCR-ABL1. We devised a bitopic inhibitor of BCR-ABL1, DasatiLink-1, based on the linking of dasatinib and asciminib by a flexible tether whose length (41 heavy atoms) was close to that of RapaLink-1 (39 heavy atoms) (Fig. 3B).

We characterized the interaction between DasatiLink-1 and its target using *in vitro* biochemical assays. Treatment of purified BCR-ABL1 kinase domain with dasatinib or asciminib caused marked (> 0.1 ppm) nuclear magnetic resonance (NMR) chemical shift differences in residues involved in binding to the monomeric inhibitors (fig. S8, A and B), consistent with previous reports (55, 56, 58). The NMR spectrum observed in the presence of Dasatilink-1 closely matched the spectrum observed with a mixture of the two non-linked inhibitors (fig. S8, A and B), suggesting that the linked inhibitor simultaneously binds to both sites and that the tether does not prevent binding to either site.

We hypothesized that DasatiLink-1 might require an allosteric foothold to achieve high occupancy of the BCR-ABL1 kinase domain, and we tested this hypothesis using a pulldown assay for ATP-site availability (59). We confirmed that the assay recapitulated a biochemical IC_50_ of < 1 nM for dasatinib (60), which was unaffected by inclusion of 100-fold excess of the allosteric inhibitor asciminib (fig. S8C). However, addition of excess asciminib impaired the ability of DasatiLink-1 to occupy the ATP-site, likely resulting from a loss of avidity following steric occlusion of the allosteric pocket (fig. S8C). This indicates that Dasatilink-1 relies on both the orthosteric and allosteric sites for binding, suggesting that it might also exhibit the enhanced selectivity often observed in bitopic inhibitors (61). Together, these biochemical data validate DasatiLink-1 as a bitopic inhibitor with physicochemical properties beyond standard drug design limits (1–3), and we reasoned that the molecule’s linked composition might allow it to be assisted into the cell by IFITMs.

Returning to our K562 CRISPRi/a models, which are patient-derived BCR-ABL1 mutant CML cells, we characterized the effect of IFITM expression on the ability of DasatiLink-1 to inhibit intracellular BCR-ABL1 signaling. Similar to RapaLink-1 (Fig. 1, E and F), CRISPRi and CRISPRa perturbation of IFITM expression resulted in a combined 8.9-fold modulation of DasatiLink-1 cellular potency (Fig. 3, C and D). We also probed the capacity of DasatiLink-1 to engage intracellular BCR-ABL1 by measuring pharmacodynamic markers of inhibition. Consistent with an IFITM-assisted uptake mechanism, *IFITM1–3* triple knockdown reduced the ability of DasatiLink-1 to inhibit phospho-BCR-ABL1^Y245^ and phospho-STAT5^Y694^, which are known BCR-ABL1 substrates (Fig. 3E). The maximal inhibition observed for DasatiLink-1 in the TriNegCtrl sg conditions at 8 and 24 h was not ever reached in the *IFITM1–3* triple knockdown conditions, likely due to lower intracellular concentrations of compound resulting from decreased uptake. The inhibition kinetics we observed for the negative control treatment, requiring multiple hours for maximal inhibition at a nanomolar concentration (Fig. 3E), were also exhibited by RapaLink-1 (Fig. 1D).

We anticipated that DasatiLink-1, akin to RapaLink-1, was likely to be selective for its target as a result of its multivalent binding mechanism – only BCR-ABL1 kinase domain contains binding sites for both of its linked components. We assessed DasatiLink-1’s kinome-wide selectivity in live cells using a promiscuous kinase occupancy probe, XO44 (62), with which kinase active-site occupancy can be determined through competitive activity-based protein profiling (63). In contrast to an unlinked control (a 1:1 mixture of dasatinib and asciminib) at equimolar concentration, which competed with XO44 for labeling of numerous known dasatinib targets (62), pretreatment with DasatiLink-1 resulted in observable intracellular occupancy of only ABL1 (Fig. 3F, data file S6). This single kinase specificity extended over a 100-fold concentration range up to 1 μM, the same range over which the unlinked control demonstrated dose-responsive occupancy of numerous off-targets (Fig. 3G, data file S6). These data suggest that target selectivity can be conferred by two-site binding, analogous to RapaLink-1’s selectivity for MTOR complex 1 (7, 24, 25).

## BisRoc-1 analogs reveal linker length dependency of IFITM-assisted cellular uptake

To further examine the breadth of linked chemotypes that might be assisted by IFITMs, we designed, synthesized, and characterized a new linked molecular glue inhibitor based on the natural product rocaglamide. Rocaglamide clamps the EIF4A1 helicase to 5’ untranslated regions (UTRs) of target mRNAs to inhibit the translation of downstream sequences (64). The crystal structure of the complex of rocaglamide, EIF4A1, and polypurine RNA (65) revealed that the molecule’s amide points toward free solvent, near a symmetry mate (fig. S9A). We reasoned that dimerization of rocaglamide through its amide position could be a chemically tractable means to simultaneously engage two proximal EIF4A1-RNA complexes within the cell. We designed a molecule, BisRoc-1 (fig. S9B), that links two rocaglamide monomers together with a linker length (35 heavy atoms) exceeding the distance separating two rocaglamide binding sites in the crystal structure (fig. S9A). Similar to RapaLink-1 and DasatiLink-1, CRISPRi and CRISPRa perturbation of IFITM expression resulted in a combined 6.2-fold modulation in cellular activity of BisRoc-1 (fig. S9, C and D). Additionally, we evaluated the relationship between linker length and IFITM assistance by examining an analog series consisting of BisRoc-1 (PEG11), BisRoc-2 (PEG4), BisRoc-3 (PEG2), and rocaglamide (no linker) (fig. S9E). We treated our K562 CRISPRi and CRISPRa cells with these inhibitors and evaluated differences in potency resulting from IFITM expression modulation, as measured by half-maximal inhibitory concentration (IC_50_) shift in a cell viability assay (fig. S9, E and F, and data file S7). This revealed a pattern in which longer linker lengths correlated with greater IFITM assistance. Combined, these data suggest the general feasibility of retaining cell permeability despite increased pharmacophore size, polarity, and flexibility in the context of linked chemotypes described herein.

## An expanded chemical space for cell permeable molecules

**Fig. 4.**
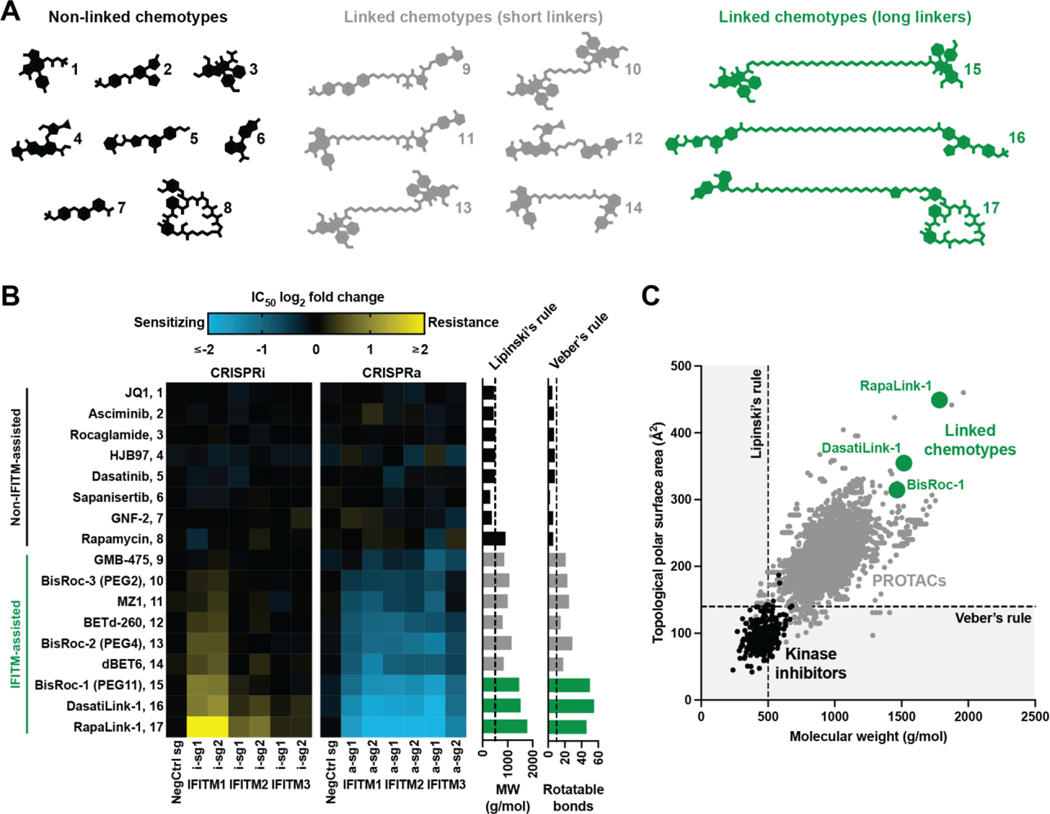
IFITMs assist the cellular activity of diverse linked chemotypes. (**A**) Heavy atom skeletons of compounds assessed for IFITM assistance (see also fig. S10 for chemical structures). Compounds were categorized as non-linked chemotypes (compounds 1–8, black), linked chemotypes with short linkers (compounds 9–14, gray), or linked chemotypes with long linkers (compounds 15–17, green). (**B**) Chemical-genetic interaction map of inhibitors in (A) with *IFITM1*, *IFTM2*, and *IFITM3*. Potency, as measured by dose-response IC_50_ in a cell viability assay (see also Fig. 1F, Fig. 3D, or fig. S9D for example source data), was normalized to that of non-sgRNA-expressing K562 CRISPRi or CRISPRa cells. Physicochemical properties, including molecular weight (MW) and number of rotatable bonds, with their respective traditional thresholds for drug-likeness are indicated (right). Data represent means of three biological replicates. (**C**) Map of chemical space populated by 304 kinase inhibitors in clinical development (black), 3270 PROTACs reported in the literature (gray), and 3 linked chemotypes described herein (green). Boundaries represent traditional guidelines for drug-likeness.

Given the ubiquitous presence of IFITMs in cells, we hypothesized that the cellular uptake of other linked inhibitors in the literature could also be assisted by IFITMs. While not generally as large as the linked chemotypes described above, PROTACs are likewise composed of two chemical entities covalently attached by a flexible tether (16). Thus, we included four PROTACs (GMB-475, MZ1, BETd-260, and dBET6) and their non-linked parent inhibitors in an expanded survey of chemical-genetic interactions with IFITMs (Fig. 4A, fig. S10, and table S1). These compounds were evaluated in the same IFITM dependency analysis as the BisRoc linker series (Fig. 4B and data file S7). Using RapaLink-1 as a chemical benchmark, we observed that *IFITM1*, *IFITM2*, and *IFITM3* overexpression sensitized cells to linked chemotypes (Fig. 4B; compounds 9-17). The inverse finding, resistance to linked chemotypes, resulted from gene knockdown (Fig. 4B). The trend observed in the BisRoc series was corroborated across the 9 bivalent molecules tested (Fig. 4B): the magnitudes of chemical-genetic interactions correlated with linker length which is reflected in inhibitor size (molecular weight) and flexibility (number of rotatable bonds). Linked chemotypes with long linkers were more IFITM-assisted than linked chemotypes with short linkers, and non-linked chemotypes (Fig. 4B; compounds 1-8) were not observed to be assisted by IFITMs (Fig. 4B). Despite their cellular activities, the physicochemical properties of the linked chemotypes largely violate Lipinski’s (1) and Veber’s (2) classic guidelines (Fig. 4, A to C, and table S1), raising the need for a revised drug design framework that considers IFITM-assisted uptake and other cellular import processes.

## Discussion

Through a combination of functional genomics and chemical methods, we uncovered an endogenous pathway involving IFITMs that in our data promotes the cellular uptake of diverse linked chemotypes. With the clinical advancement of a dimeric immunophilin ligand (23), PROTACs (16), and a RapaLink-1 derivative (26), the notion of ‘drug-like’ is continually being revised. As evidence, the chemical space (66) populated by an ever-expanding set of linked preclinical compounds in the literature ventures beyond that occupied by lead inhibitors developed under traditional guidelines (Fig. 4C) (1–3). Here, we identify IFITM-assisted cellular uptake as one of the mechanisms by which linked inhibitors are able to break previously established rules surrounding drug-likeness. We anticipate that our findings will inform the uptake optimization of emerging classes of bivalent molecules (PROTACs, Syn-TEFs, RIBOTACs, PHICS, DUBTACs, and others) (4–11) and enable the design of cell permeable therapeutics that bridge distal binding sites on solitary targets or multi-target complexes.

## Supplementary Material

Supplementary materials text, figures, tables

Data file S1 CRISPRi sgRNAs

Data file S2 CRISPRi genes

Data file S3 CRISPRa sgRNAs

Data file S4 CRISPRa genes

Data file S5 Sensitivity-expression correlations.xlsx

Data file S6 XO44 kinase profiling

Data file S7 Cell viability IC50s

## Data Availability

All data are available in the manuscript or the supplementary materials. Scripts implementing analyses are available at https://github.com/dwassarman, https://github.com/mhorlbeck, and Zenodo (67, 68). Materials are available upon request to the corresponding authors with a signed material transfer agreement.
