# Supplementary material for: IFITM proteins assist cellular uptake of diverse linked chemotypes
Source: Science. Author manuscript; Available in PMC 2023 Feb 13. (PMC9924227; doi:10.1126/science.abl5829)
Supplement: Supplementary materials text, figures, tables [file NIHMS1857200-supplement-Supplementary_materials_text__figures__tables.pdf]

Chemical Synthesis

Nuclear magnetic resonance (NMR) spectra were recorded on a Bruker spectrometer at 400 MHz or on a Bruker spectrometer at 600 MHz. Chemical shifts were reported as parts per million (ppm) from solvent references. Liquid chromatography-mass spectrometry (LC-MS) was performed on a Waters Xevo G2-XS QTof (0.6 mL/min) using an ACQUITY UPLC BEH C18 column (Waters) and a water/acetonitrile gradient (0.05% formic acid) using Optima LC-MS grade solvents (Fisher Scientific). All other solvents (Fisher Scientific, Millipore Sigma) and commercially available reagents were used without further purification. Analytical thin-layer chromatography was performed with silica gel 60 F254 glass plates (Millipore Sigma). Flash chromatography was performed with RediSep Rf normal-phase silica flash columns using a CombiFlash Rf+ (Teledyne ISCO). Preparative high-performance liquid chromatography (HPLC) was performed on an AutoPurification System using an XBridge BEH C18 OBD Prep Column (Waters) or a CombiFlash EZ Prep using a RediSep C18 Prep HPLC Column (Teledyne ISCO) with a water/acetonitrile gradient (0.1% formic acid or 0.1% trifluoroacetic acid). Microwave reactions were performed using a Discover SP (CEM).

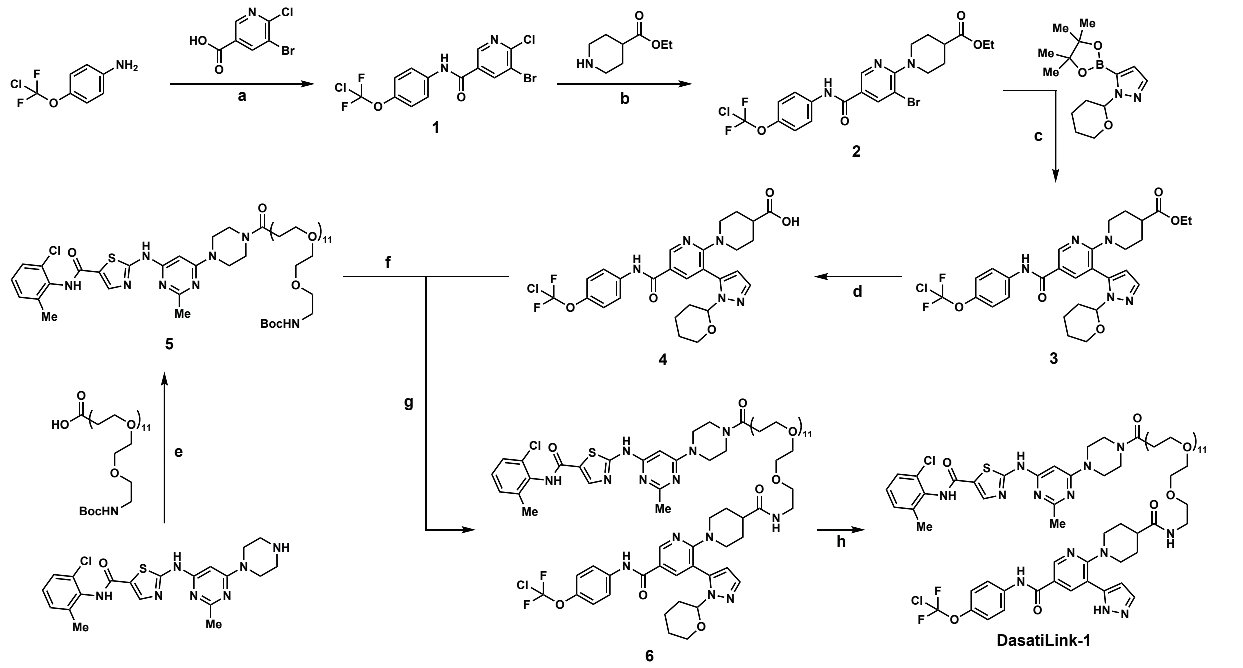

Reagents and conditions. (a) HATU, DIPEA, DMF, rt, 80%. (b) DIPEA, IPA, 140 °C, 91%. (c) K_3PO_4, $Pd(PPh_3)_4$, toluene, 110 °C, 40%. (d) $LiOH{\cdot}H_2O$, H_2O, MeOH, 98%. (e) HATU, DIPEA, DMF, rt, 87%. (f) TFA, CH_2Cl_2, rt. (g) HATU, DIPEA, DMF, rt, 71% over two steps. (h) TFA, CH_2Cl_2, rt, 72%. Abbreviations: HATU, 1-[bis(dimethylamino)methylene]-1*H*-1,2,3-triazolo[4,5-b]pyridinium 3-oxide hexafluorophosphate; DIPEA, *N*,*N*-diisopropylethylamine; DMF, *N*,*N*-dimethylformamide; IPA, isopropyl alcohol; TFA, trifluoroacetic acid.

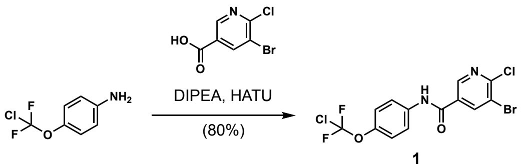

Compound 1. To a mixture of 4-(chlorodifluoromethoxy)aniline (683 mg, 3.53 mmol) and 5-bromo-6-chloropicolinic acid (1001 mg, 4.23 mmol) in *N*,*N*-dimethylformamide (17.6 mL) was added *N*,*N*-diisopropylethylamine (614 µL, 3.53 mmol). The solution was cooled in an ice-water bath before the addition of 1-[bis(dimethylamino)methylene]-1*H*-1,2,3-triazolo[4,5-b]pyridinium 3-oxide hexafluorophosphate (1744 mg, 4.59 mmol) and stirred at room temperature overnight. The mixture was partitioned between ethyl acetate and water and the organic layer was washed with water (4×) and brine (2×), dried over sodium sulfate, filtered, and concentrated *in vacuo*. The crude was purified by flash chromatography over silica gel eluting with a gradient from 0% ethyl acetate-hexanes to 20% ethyl acetate-hexanes to afford compound 1 (1162 mg, 2.82 mmol, 80% yield) as a beige solid.

^{1}H NMR (400 MHz, DMSO-d_6) δ 10.69 (s, 1H), 8.92 (d, *J* = 2.2 Hz, 1H), 8.73 (d, *J* = 2.1 Hz, 1H), 7.87 (d, *J* = 9.1 Hz, 2H), 7.39 (d, *J* = 9.2 Hz, 2H).
^{13}C NMR (100 MHz, DMSO-d_6) δ 161.8, 151.9, 147.8, 145.4, 141.8, 137.7, 130.9, 124.9 (t, $J_{C\text{-}F}$ = 287.1 Hz), 122.0 (2C), 121.7 (2C), 119.3.
^{19}F NMR (376 MHz, DMSO-d_6) δ -24.8.
HRMS (*m/z*): calculated for $C_{13}H_8BrCl_2F_2N_2O_2^+$ [M + H]$^+$ 410.9109, found 410.9123.
TLC: R_f = 0.4 (20% ethyl acetate-hexanes, UV).

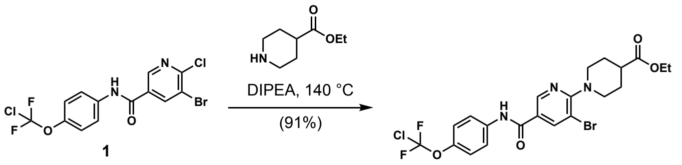

Compound 2. To a mixture of compound 1 (1157 mg, 2.81 mmol) and ethyl 4-piperidinecarboxylate (0.563 mL, 3.65 mmol) in isopropanol (2.81 mL) was added *N*,*N*-diisopropylethylamine (2.45 mL, 14.0 mmol) in a microwave reaction vial. The reaction was heated in a microwave reactor at 140 °C for 1 h. The mixture was cooled to room temperature and diluted with ethyl acetate. The organic layer was washed with water (4×), 1 N HCl (2×), and brine (2×), dried over sodium sulfate, filtered, and concentrated *in vacuo*. The crude was purified by flash chromatography over silica gel eluting with a gradient from 0% ethyl acetate-hexanes to 30% ethyl acetate-hexanes to afford compound 2 (1354 mg, 2.54 mmol, 91% yield) as a white solid.

^{1}H NMR (400 MHz, DMSO-d_6) δ 10.40 (s, 1H), 8.78 (d, *J* = 2.1 Hz, 1H), 8.44 (d, *J* = 2.1 Hz, 1H), 7.86 (d, *J* = 9.2 Hz, 2H), 7.35 (d, *J* = 9.2 Hz, 2H), 4.09 (q, *J* = 7.1 Hz, 2H), 3.98 – 3.75 (m, 2H), 3.08 – 2.87 (m, 2H), 2.66 – 2.53 (m, 1H), 2.04 – 1.88 (m, 2H), 1.82 – 1.62 (m, 2H), 1.20 (t, *J* = 7.1 Hz, 3H).

^{13}C NMR (100 MHz, DMSO-d_6) δ 174.1, 162.6, 160.5, 146.4, 145.1, 141.5, 138.2, 125.0 (t, J_{C-F} = 287.0 Hz), 124.2, 121.9 (2C), 121.5 (2C), 109.5, 59.9, 48.3 (2C), 40.0, 27.7 (2C), 14.1.
^{19}F NMR (376 MHz, DMSO-d_6) δ -24.7.
HRMS (*m/z*): calculated for $C_{21}H_{22}BrClF_2N_3O_4^+$ $[M + H]^+$ 532.0445, found 532.0473
TLC: R_f = 0.4 (30% ethyl acetate-hexanes, UV)

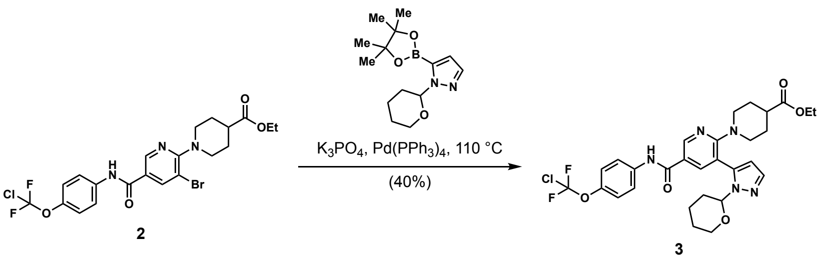

Compound 3. To a mixture of compound 2 (600 mg, 1.13 mmol), 1-(tetrahydro-2*H*-pyran-2-yl)-1*H*-pyrazole-4-boronic acid pinacol ester (407 mg, 1.46 mmol), and K_3PO_4 (717 mg, 3.38 mmol) in toluene (1.13 mL) was added $Pd(PPh_3)_4$ (65 mg, 0.056 mmol). The reaction was sparged with argon for 5 min before stirring at 110 °C for 2 h. The mixture was diluted with ethyl acetate and the organic layer was washed with saturated sodium bicarbonate, water (2×), and brine (2×), dried over sodium sulfate, filtered, and concentrated *in vacuo*. The crude was purified by flash chromatography over silica gel eluting with a gradient from 0% ethyl acetate-hexanes to 50% ethyl acetate-hexanes to afford compound 3 (273 mg, 0.452 mmol, 40% yield) as a white solid.

^{1}H NMR (400 MHz, DMSO-d_6) δ 10.34 (s, 1H), 8.84 (d, *J* = 2.4 Hz, 1H), 8.09 (d, *J* = 2.5 Hz, 1H), 7.87 (d, *J* = 9.2 Hz, 2H), 7.66 (d, *J* = 1.7 Hz, 1H), 7.35 (d, *J* = 8.7 Hz, 2H), 6.48 (d, *J* = 1.8 Hz, 1H), 5.16 (dd, *J* = 9.8, 2.4 Hz, 1H), 4.04 (q, *J* = 7.1 Hz, 2H), 3.90 – 3.74 (m, 1H), 3.73 – 3.51 (m, 2H), 3.38 – 3.29 (m, 1H), 2.94 – 2.70 (m, 2H), 2.60 – 2.43 (m, 1H), 2.43 – 2.24 (m, 1H), 2.04 – 1.91 (m, 1H), 1.90 – 1.80 (m, 1H), 1.79 – 1.66 (m, 2H), 1.63 – 1.53 (m, 1H), 1.53 – 1.38 (m, 4H), 1.16 (t, *J* = 7.1 Hz, 3H).
^{13}C NMR (100 MHz, DMSO-d_6) δ 174.1, 163.6, 160.2, 148.3, 145.0, 140.7, 140.0, 139.5, 138.4, 125.0 (t, J_{C-F} = 287.0 Hz), 121.8 (2C), 121.5 (2C), 120.9, 112.5, 106.6, 83.9, 66.7, 59.9, 47.0, 46.8, 40.0, 29.1, 27.5, 27.3, 24.5, 22.1, 14.1.
^{19}F NMR (376 MHz, DMSO-d_6) δ -24.7.
HRMS (*m/z*): calculated for $C_{29}H_{33}ClF_2N_5O_5^+$ $[M + H]^+$ 604.2133, found 604.2150
TLC: R_f = 0.5 (50% ethyl acetate-hexanes, UV)

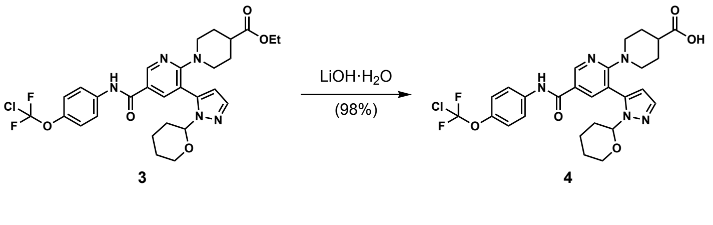

Compound 4. To a mixture of compound 3 (60 mg, 0.099 mmol) in methanol (2 mL) and water (0.2 mL) was added lithium hydroxide monohydrate (12.5 mg, 0.298 mmol). The reaction was

stirred at room temperature overnight. The mixture was concentrated *in vacuo* and partitioned between ethyl acetate and 5% citric acid. The aqueous layer was extracted with ethyl acetate (3×), and the combined organics were washed with water (4×) and brine (2×), dried over sodium sulfate, filtered, and concentrated *in vacuo*. The crude was purified by flash chromatography over silica gel eluting with a gradient from 0% methanol-ethyl acetate to 5% methanol-ethyl acetate to afford compound 4 (56 mg, 0.097 mmol, 98% yield) as a white solid.

^{1}H NMR (400 MHz, DMSO-d_6) δ 12.19 (s, 1H), 10.33 (s, 1H), 8.84 (d, J = 2.4 Hz, 1H), 8.09 (d, J = 2.4 Hz, 1H), 7.86 (d, J = 9.1 Hz, 2H), 7.66 (d, J = 1.7 Hz, 1H), 7.35 (d, J = 8.9 Hz, 2H), 6.48 (d, J = 1.8 Hz, 1H), 5.16 (dd, J = 9.7, 2.4 Hz, 1H), 3.88 – 3.77 (m, 1H), 3.70 – 3.53 (m, 2H), 3.38 – 3.33 (m, 1H), 2.90 – 2.72 (m, 2H), 2.46 – 2.38 (m, 1H), 2.38 – 2.27 (m, 1H), 2.02 – 1.91 (m, 1H), 1.91 – 1.80 (m, 1H), 1.78 – 1.65 (m, 2H), 1.64 – 1.52 (m, 1H), 1.52 – 1.39 (m, 4H).
^{13}C NMR (100 MHz, DMSO-d_6) δ 175.8, 163.6, 160.3, 148.3, 145.0, 140.7, 140.0, 139.4, 138.4, 125.0 (t, J_{C-F} = 286.9 Hz), 121.8 (2C), 121.5 (2C), 120.8, 112.4, 106.6, 83.9, 66.7, 47.1, 46.9, 40.0, 29.1, 27.6, 27.4, 24.5, 22.1.
^{19}F NMR (376 MHz, DMSO-d_6) δ -24.7.
HRMS (*m/z*): calculated for $C_{27}H_{29}ClF_2N_5O_5^+$ $[M + H]^+$ 576.1820, found 576.1816
TLC: R_f = 0.5 (5% methanol-ethyl acetate, UV)

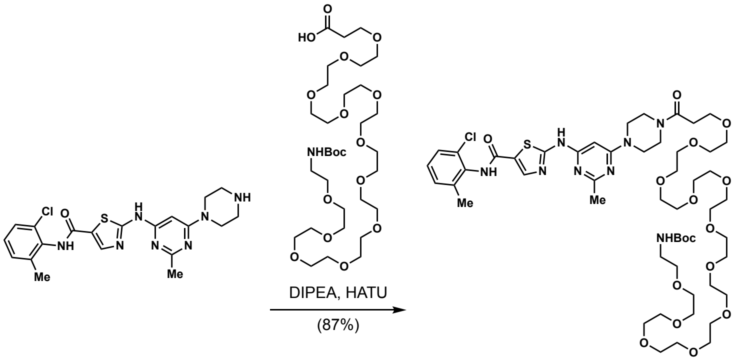

5

Compound 5. To a mixture of *N*-deshydroxyethyl dasatinib (100 mg, 0.225 mmol) and *t*-Boc-*N*-amido-PEG12-acid (161.69 mg, 0.225 mmol) in *N*,*N*-dimethylformamide (1.13 mL) was added *N*,*N*-diisopropylethylamine (118 µL, 0.676 mmol). The solution was cooled in an ice-water bath before the addition of 1-[bis(dimethylamino)methylene]-1*H*-1,2,3-triazolo[4,5-b]pyridinium 3-oxide hexafluorophosphate (94 mg, 0.248 mmol) and stirred at room temperature overnight. The mixture was partitioned between ethyl acetate and water and the organic layer was washed with saturated sodium bicarbonate (3×), water (4×) and brine (2×), dried over sodium sulfate, filtered, and concentrated *in vacuo*. The crude was purified by flash chromatography over silica gel eluting with a gradient from 0% methanol-dichloromethane to 20% methanol-dichloromethane to afford compound 5 (223 mg, 0.195 mmol, 87% yield) as a pale yellow semisolid.

^{1}H NMR (600 MHz, DMSO-d_6) δ 11.50 (s, 1H), 9.88 (s, 1H), 8.22 (s, 1H), z7 – 7.34 (m, 1H), 7.34 – 7.19 (m, 2H), 6.81 – 6.63 (m, 1H), 6.06 (s, 1H), 3.65 (t, J = 6.6 Hz, 2H), 3.63 – 3.52 (m, 8H), 3.52 – 3.42 (m, 44H), 3.37 (t, J = 6.1 Hz, 2H), 3.12 – 2.97 (m, 2H), 2.62 (t, J = 6.6 Hz, 2H), 2.42 (s, 3H), 2.24 (s, 3H), 1.37 (s, 9H).

^{13}**C NMR** (151 MHz, DMSO-d_6) δ 169.1, 165.2, 162.5, 162.2, 159.9, 157.0, 155.6, 140.8, 138.8, 133.5, 132.4, 129.0, 128.2, 127.0, 125.8, 82.7, 77.6, 70.1 – 69.3 (m, 22C), 69.2, 66.8, 44.3, 43.5, 43.2, 40.5, 39.5, 32.9, 28.2 (3C), 25.6, 18.3.
HRMS (*m/z*): calculated for $C_{52}H_{84}ClN_8O_{16}S^+$ $[M + H]^+$ 1143.5409, found 1143.5419
TLC: R_f = 0.5 (20% methanol-dichloromethane)

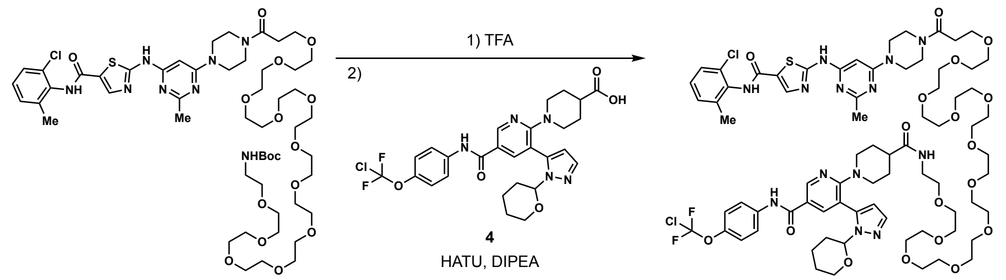

Compound 6. To a mixture of compound 5 (100 mg, 0.0874 mmol) in dichloromethane (0.874 mL) was added trifluoroacetic acid (0.874 mL). The solution was stirred at room temperature for 1 h before concentrating *in vacuo* to afford a pale yellow semisolid that was used directly in the next step. To a mixture of the crude amine, trifluoroacetic acid salt and compound 4 (55 mg, 0.0961 mmol) in *N*,*N*-dimethylformamide (0.874 mL) was added *N*,*N*-diisopropylethylamine (46 µL, 0.262 mmol). The solution was cooled in an ice-water bath before the addition of 1-[bis(dimethylamino)methylene]-1*H*-1,2,3-triazolo[4,5-b]pyridinium 3-oxide hexafluorophosphate (37 mg, 0.0961 mmol) and stirred at room temperature overnight. The mixture was partitioned between ethyl acetate and water and the organic layer was washed with water (4×) and brine (2×), dried over sodium sulfate, filtered, and concentrated *in vacuo*. The crude was purified by flash chromatography over silica gel as follows: the crude was dry loaded into silica gel and an initial elution with ethyl acetate was made to remove an impurity. Upon switching to a methanol-dichloromethane solvent system, the desired product immediately eluted with additional impurities from the column. Fractions containing the desired product were then re-subjected to flash chromatography over silica gel eluting with a gradient from 0% methanol-dichloromethane to 20% methanol-dichloromethane to afford compound 6 (99 mg, 0.0618 mmol, 71% yield over two steps) as a pale yellow solid.

^{1}**H NMR** (600 MHz, DMSO-d_6) δ 11.50 (s, 1H), 10.33 (s, 1H), 9.88 (s, 1H), 8.84 (d, *J* = 2.4 Hz, 1H), 8.22 (s, 1H), 8.08 (d, *J* = 2.4 Hz, 1H), 7.90 – 7.83 (m, 2H), 7.83 – 7.75 (m, 1H), 7.65 (d, *J* = 1.7 Hz, 1H), 7.43 – 7.37 (m, 1H), 7.34 (d, *J* = 9.2 Hz, 2H), 7.31 – 7.19 (m, 2H), 6.47 (d, *J* = 1.7 Hz, 1H), 6.06 (s, 1H), 5.16 (dd, *J* = 9.8, 2.5 Hz, 1H), 3.85 – 3.75 (m, 1H), 3.73 – 3.66 (m, 2H), 3.65 (t, *J* = 6.6 Hz, 2H), 3.62 – 3.51 (m, 8H), 3.51 – 3.46 (m, 44H), 3.37 (t, *J* = 6.0 Hz, 2H), 3.33 – 3.29 (m, 1H), 3.20 – 3.09 (m, 2H), 2.80 – 2.65 (m, 2H), 2.62 (t, *J* = 6.6 Hz, 2H), 2.42 (s, 3H), 2.38 – 2.26 (m, 2H), 2.24 (s, 3H), 1.99 – 1.85 (m, 2H), 1.61 – 1.40 (m, 7H).
^{13}**C NMR** (151 MHz, DMSO-d_6) δ 174.0, 169.1, 165.2, 163.6, 162.5, 162.2, 160.3, 159.9, 157.0, 148.3, 145.0, 140.8, 140.8, 140.0, 139.4, 138.8, 138.4, 133.5, 132.4, 129.0, 128.2, 127.0, 125.8, 125.0 (t, $J_{C\text{-}F}$ = 286.9 Hz), 121.9 (2C), 121.5 (2C), 120.8, 112.4, 106.6, 84.0, 82.7, 70.1 – 69.3 (m, 22C), 69.1, 66.8, 66.7, 47.4, 47.1, 44.3, 43.5, 43.2, 41.5, 40.5, 38.4, 32.9, 29.0, 28.1, 27.9, 25.6, 24.5, 22.1, 18.3.

^{19}F NMR (564 MHz, DMSO-d_6) δ -24.7.
HRMS (*m/z*): calculated for $C_{74}H_{102}Cl_2F_2N_{13}O_{18}S^+$ [M + H]$^+$ 1600.6526, found 1600.6644.
TLC: R_f = 0.0 (ethyl acetate), R_f = 0.4 (20% methanol-dichloromethane)

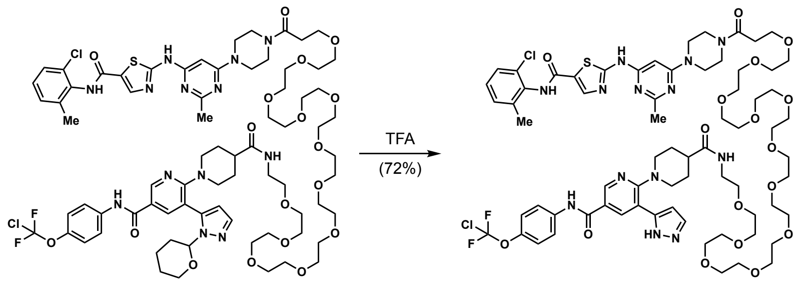

DasatiLink-1. To a mixture of compound 9 (47 mg, 0.0293 mmol) in dichloromethane (0.293 mL) was added trifluoroacetic acid (0.293 mL). The solution was stirred at room temperature for 6 h before concentrating *in vacuo*. The residue was partitioned between ethyl acetate and saturated sodium bicarbonate. The aqueous layer was extracted with ethyl acetate (3×) and the combined organics were washed with brine (2×), dried over sodium sulfate, filtered, and concentrated *in vacuo*. The crude was purified by flash chromatography over silica gel eluting with a gradient from 0% methanol-dichloromethane to 20% methanol-dichloromethane. Fractions containing the desired product were combined, concentrated *in vacuo*, and further purified by HPLC to afford DasatiLink-1 (32 mg, 0.0211 mmol, 72% yield) as a white solid.

^{1}H NMR (600 MHz, DMSO-d_6) δ 13.02 (s, 1H), 11.50 (s, 1H), 10.39 (s, 1H), 9.88 (s, 1H), 8.74 (d, J = 2.4 Hz, 1H), 8.34 (s, 1H), 8.22 (s, 1H), 7.97 – 7.52 (m, 4H), 7.43 – 7.37 (m, 1H), 7.34 (d, J = 8.7 Hz, 2H), 7.31 – 7.22 (m, 2H), 6.66 (s, 1H), 6.06 (s, 1H), 3.71 – 3.61 (m, 4H), 3.61 – 3.52 (m, 8H), 3.52 – 3.44 (m, 44H), 3.39 (t, J = 6.0 Hz, 2H), 3.21 – 3.15 (m, 2H), 2.75 – 2.67 (m, 2H), 2.62 (t, J = 6.6 Hz, 2H), 2.42 (s, 3H), 2.32 – 2.25 (m, 1H), 2.24 (s, 3H), 1.69 – 1.55 (m, 4H).
^{13}C NMR (151 MHz, DMSO-d_6) δ 174.3, 169.1, 165.2, 164.1, 162.5, 162.2, 160.9, 159.9, 157.0, 148.4, 146.5, 144.9, 140.8, 138.8, 138.5, 137.6, 133.5, 132.4, 129.7, 129.0, 128.2, 127.0, 125.8, 125.0 (t, $J_{C\text{-}F}$ = 286.9 Hz), 122.1, 121.9 (2C), 121.5 (2C), 118.2, 103.5, 82.8, 70.3 – 69.3 (m, 22C), 69.1, 66.8, 48.4 (2C), 44.4, 43.5, 43.2, 41.7, 40.5, 38.5, 32.9, 28.1 (2C), 25.6, 18.3.
^{19}F NMR (564 MHz, DMSO-d_6) δ -24.7.
HRMS (*m/z*): calculated for $C_{69}H_{94}Cl_2F_2N_{13}O_{17}S^+$ [M + H]$^+$ 1516.5951, found 1516.6038
TLC: R_f = 0.5 (20% methanol-dichloromethane)

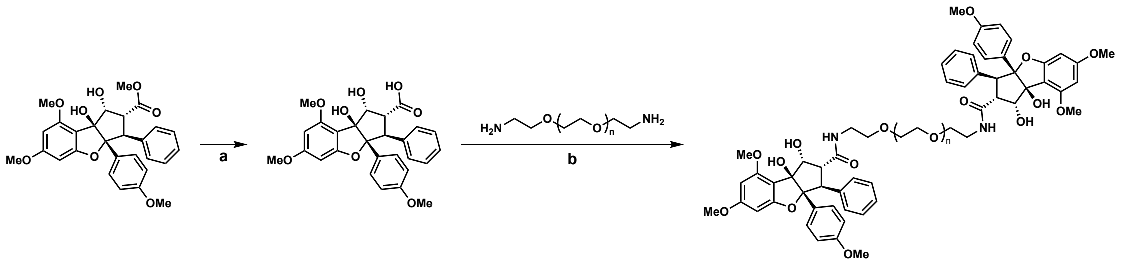

BisRoc-1, 2, 3

Reagents and conditions. (a) Aqueous LiOH, THF, 60 °C, 93%. (b) HATU, DIPEA, DMF, rt, 63-81%. Abbreviations: HATU, 1-[bis(dimethylamino)methylene]-1*H*-1,2,3-triazolo[4,5-b]pyridinium 3-oxide hexafluorophosphate; DIPEA, *N*,*N*-diisopropylethylamine; DMF, *N*,*N*-dimethylformamide.

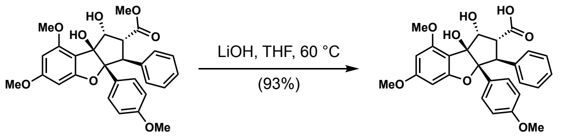

Rocagloic acid. This protocol was adapted from Lajkiewicz *et al.*, *J. Am. Chem. Soc.* **136**, 2659–2664 (2014). To a mixture of aglafoline (73 mg, 0.148 mmol) in THF (3.7 mL) was added 0.1 M lithium hydroxide in water (3.7 mL, 0.371 mmol). The reaction was stirred at 60 °C for 5 h. After allowing the reaction to cool to room temperature, the mixture was partitioned between dichloromethane and 5% citric acid in water. The aqueous layer was extracted with dichloromethane (4×) and the combined organics were washed with brine (2×), dried over sodium sulfate, filtered, and concentrated *in vacuo*. The crude was purified by flash chromatography over silica gel eluting with a gradient from 0% methanol-dichloromethane to 20% methanol-dichloromethane to afford rocagloic acid (66 mg, 0.138 mmol, 93% yield) as a white solid.

^{1}H NMR (400 MHz, DMSO-d_6) δ 12.05 (s, 1H), 7.08 – 6.92 (m, 5H), 6.88 (d, *J* = 7.2 Hz, 2H), 6.58 (d, *J* = 9.0 Hz, 2H), 6.27 (d, *J* = 1.9 Hz, 1H), 6.11 (d, *J* = 2.0 Hz, 1H), 5.01 (s, 1H), 4.95 (s, 1H), 4.67 (d, *J* = 5.7 Hz, 1H), 4.10 (d, *J* = 14.0 Hz, 1H), 3.79 (dd, *J* = 14.2, 5.7 Hz, 1H), 3.78 (s, 3H), 3.74 (s, 3H), 3.60 (s, 3H).
^{13}C NMR (100 MHz, DMSO-d_6) δ 171.2, 162.6, 160.5, 157.8, 157.4, 138.7, 128.7, 128.7 (2C), 127.7 (2C), 127.3 (2C), 125.7, 111.8 (2C), 108.4, 101.4, 93.3, 91.8, 88.4, 78.8, 55.5, 55.4, 54.7, 54.7, 50.9.
HRMS (*m/z*): calculated for $C_{27}H_{27}O_8^+$ [M + H]$^+$ 479.1700, found 479.1706
TLC: R_f = 0.6 (20% methanol-dichloromethane)

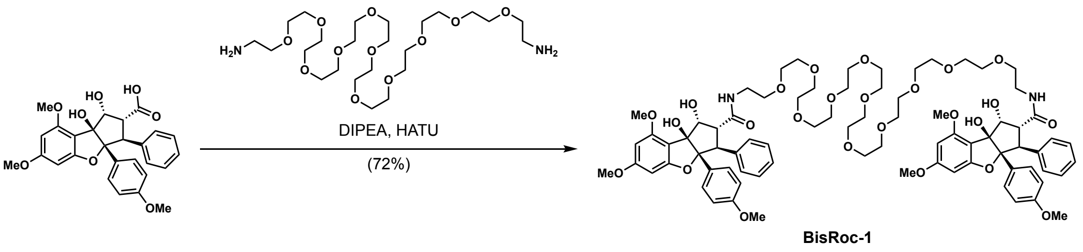

BisRoc-1. To a mixture of rocagloic acid (30 mg, 0.0627 mmol) and amino-PEG11-amine (17.1 mg, 0.0314 mmol) in *N*,*N*-dimethylformamide (0.627 mL) was added *N*,*N*-diisopropylethylamine (55 µL, 0.313 mmol). The solution was cooled in an ice-water bath before the addition of 1-[bis(dimethylamino)methylene]-1*H*-1,2,3-triazolo[4,5-b]pyridinium 3-oxide hexafluorophosphate (26 mg, 0.0684 mmol) and stirred at room temperature overnight. The mixture was partitioned between ethyl acetate and water and the organic layer was washed with water (4×) and brine (2×), dried over sodium sulfate, filtered, and concentrated *in vacuo*. The crude was purified by flash chromatography over silica gel eluting with a gradient from 0% methanol-dichloromethane to 10% methanol-dichloromethane to afford BisRoc-1 (33 mg, 0.0225 mmol, 72% yield) as a white solid.

^{1}H NMR (400 MHz, DMSO-d_6) δ 8.39 – 8.19 (m, 2H), 7.10 – 6.99 (m, 8H), 6.99 – 6.88 (m, 6H), 6.59 (d, *J* = 8.9 Hz, 4H), 6.27 (d, *J* = 1.9 Hz, 2H), 6.10 (d, *J* = 2.0 Hz, 2H), 4.94 (s, 2H), 4.59 – 4.50 (m, 4H), 4.17 (d, *J* = 14.1 Hz, 2H), 3.85 (dd, *J* = 14.1, 5.0 Hz, 2H), 3.77 (s, 6H), 3.73 (s, 6H), 3.60 (s, 6H), 3.53 – 3.43 (m, 40H), 3.37 – 3.27 (m, 4H), 3.23 – 3.08 (m, 4H).
^{13}C NMR (100 MHz, DMSO-d_6) δ 170.3 (2C), 162.6 (2C), 160.6 (2C), 157.9 (2C), 157.4 (2C), 138.5 (2C), 128.9 (2C), 128.7 (4C), 127.9 (4C), 127.3 (4C), 125.7 (2C), 111.7 (4C), 108.5 (2C), 101.3 (2C), 93.3 (2C), 91.7 (2C), 88.4 (2C), 78.9 (2C), 69.8 (16C), 69.7 (2C), 69.6 (2C), 69.1 (2C), 55.5 (2C), 55.4 (2C), 55.3 (2C), 54.7 (2C), 50.0 (2C), 38.6 (2C).
HRMS (*m/z*): calculated for $C_{78}H_{101}N_2O_{25}^+$ $[M + H]^+$ 1465.6688, found 1465.6643
TLC: $R_f = 0.2$ (10% methanol-dichloromethane)

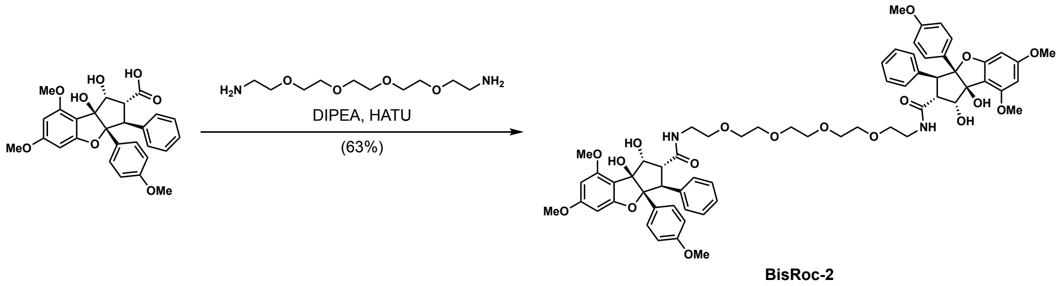

BisRoc-2. The same procedure as for BisRoc-1, using amino-PEG4-amine as starting material with scaled reagents, afforded BisRoc-2 (23 mg, 0.0199 mmol, 63% yield) as a white solid.

^{1}H NMR (400 MHz, DMSO-d_6) δ 8.40 – 8.19 (m, 2H), 7.11 – 6.99 (m, 8H), 6.99 – 6.88 (m, 6H), 6.59 (d, *J* = 9.0 Hz, 4H), 6.27 (d, *J* = 2.0 Hz, 2H), 6.10 (d, *J* = 2.0 Hz, 2H), 4.95 (s, 2H), 4.54 (d, *J* = 5.3 Hz, 4H), 4.17 (d, *J* = 14.1 Hz, 2H), 3.85 (dd, *J* = 14.2, 5.3 Hz, 2H), 3.77 (s, 6H), 3.73 (s,

6H), 3.60 (s, 6H), 3.51 (s, 4H), 3.50 – 3.47 (m, 4H), 3.47 – 3.43 (m, 4H), 3.39 – 3.26 (m, 4H), 3.22 – 3.09 (m, 4H).

^{13}C NMR (100 MHz, DMSO-d_6) δ 170.3 (2C), 162.6 (2C), 160.6 (2C), 157.9 (2C), 157.4 (2C), 138.5 (2C), 128.9 (2C), 128.7 (4C), 127.9 (4C), 127.3 (4C), 125.7 (2C), 111.7 (4C), 108.5 (2C), 101.3 (2C), 93.3 (2C), 91.7 (2C), 88.4 (2C), 78.9 (2C), 69.8 (2C), 69.7 (2C), 69.6 (2C), 69.1 (2C), 55.4 (2C), 55.4 (2C), 55.3 (2C), 54.7 (2C), 50.0 (2C), 38.6 (2C).

HRMS (*m/z*): calculated for $C_{64}H_{73}N_2O_{18}^+$ $[M + H]^+$ 1157.4853, found 1157.4843

TLC: R_f = 0.3 (10% methanol-dichloromethane)

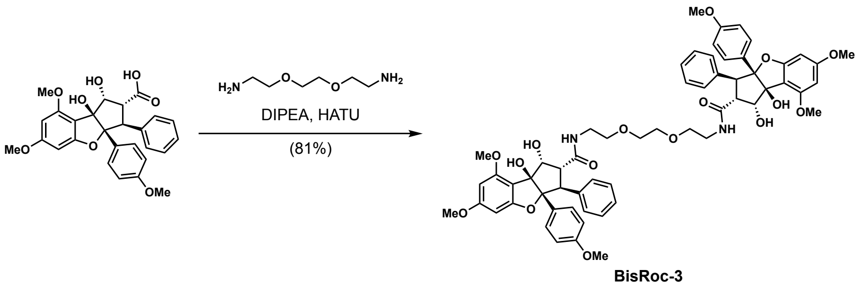

BisRoc-3

BisRoc-3. The same procedure as for BisRoc-1, using amino-PEG2-amine as starting material with scaled reagents, afforded BisRoc-3 (27 mg, 0.0253 mmol, 81% yield) as a white solid.

^{1}H NMR (400 MHz, DMSO-d_6) δ 8.47 – 8.11 (m, 2H), 7.13 – 6.99 (m, 8H), 6.99 – 6.89 (m, 6H), 6.59 (d, J = 9.0 Hz, 4H), 6.27 (d, J = 2.0 Hz, 2H), 6.10 (d, J = 2.0 Hz, 2H), 4.96 (s, 2H), 4.54 (d, J = 5.3 Hz, 4H), 4.18 (d, J = 14.1 Hz, 2H), 3.85 (dd, J = 14.1, 5.2 Hz, 2H), 3.78 (s, 6H), 3.72 (s, 6H), 3.60 (s, 6H), 3.43 (s, 4H), 3.39 – 3.26 (m, 4H), 3.22 – 3.09 (m, 4H).

^{13}C NMR (100 MHz, DMSO-d_6) δ 170.3 (2C), 162.6 (2C), 160.6 (2C), 157.9 (2C), 157.4 (2C), 138.4 (2C), 128.8 (2C), 128.7 (4C), 127.9 (4C), 127.3 (4C), 125.7 (2C), 111.7 (4C), 108.5 (2C), 101.3 (2C), 93.3 (2C), 91.7 (2C), 88.4 (2C), 78.9 (2C), 69.5 (2C), 69.1 (2C), 55.5 (2C), 55.4 (2C), 55.3 (2C), 54.7 (2C), 50.0 (2C), 38.6 (2C).

HRMS (*m/z*): calculated for $C_{60}H_{65}N_2O_{16}^+$ $[M + H]^+$ 1069.4329, found 1069.4337

TLC: R_f = 0.3 (10% methanol-dichloromethane)

Materials and Methods

Cell culture and reagents

K562 CRISPRi and CRISPRa cell lines were generated as described previously (*28*). K562 cells were maintained in RPMI medium (Gibco) supplemented with 10% (v/v) fetal bovine serum (FBS) (Avantor Seradigm), penicillin (100 U/mL, Gibco), streptomycin (100 µg/mL, Gibco), and 0.1% (v/v) Pluronic F-68 (Gibco) unless otherwise specified. HEK293T cells were maintained in DMEM medium (Gibco) supplemented with 10% (v/v) FBS (Avantor Seradigm), penicillin (100 U/mL, Gibco), and streptomycin (100 g/mL, Gibco). All cells were grown in 37 °C, 5% CO_2 stationary culture unless otherwise specified. Cell counting was performed on an Attune NxT (Thermo Fisher Scientific) or Countess II FL Automated Cell Counter (Thermo Fisher Scientific). Cells were periodically tested for mycoplasma contamination using the MycoAlert PLUS Mycoplasma Detection Kit (Lonza). Dasatinib, rapamycin, and sapanisertib were obtained from LC Laboratories. TAMRA-N_3 was obtained from BroadPharm. Asciminib, BETd-260, dBET6, GMB-475, GNF-2, HJB97, JQ1, MZ1, and rocaglamide were obtained from MedChemExpress. RapaLink-1, RapaTAMRA-PEG8, and TAMRA-PEG8-N_3 were synthesized as described previously (*7*, *27*). DasatiLink-1, BisRoc-1, BisRoc-2, and BisRoc-3 were synthesized as described herein. Compounds were stored at -20 °C as 10 mM stock solutions in dimethyl sulfoxide (DMSO) or dilutions thereof. The concentrations indicated for inhibitor combinations (eg. sapanisertib + rapamycin) represent the stoichiometric abundance of each solute individually (ie. a 1 nM sapanisertib + rapamycin treatment is equivalent to treating cells with 1 nM sapanisertib AND 1 nM rapamycin).

DNA transfections and lentivirus production

HEK293T cells were transfected with sgRNA expression vectors and standard packaging vectors (pCMV-dR8.91 and pMD2.G) using TransIT-LT1 Transfection Reagent (Mirus Bio). Lentiviral supernatant was collected 2-3 days following transfection, filtered through sterile 0.45 µm polyvinylidene difluoride filters (Millipore), and stored at -80 °C.

Genome-scale CRISPRi/a screening

Genome-scale CRISPRi/a screens were modeled after previous examples (*28*, *29*). Over the course of the screens, cells were grown in 500 mL Optimum Growth Flasks (Thomson) in 37 °C, 5% CO_2 shaking culture [1300 revolutions per minute in a Multitron Incubator (Infors HT)]. K562 CRISPRi or CRISPRa cells were transduced with the five-sgRNA/gene human CRISPRi v2 (hCRISPRi-v2) or five-sgRNA/gene human CRISPRa v2 (hCRISPRa-v2) library respectively in the presence of polybrene (8 µg/mL) (*29*). Viral transduction was tittered to maximize singly transduced cells, targeting a multiplicity of infection (MOI) ≤ 1 (percentage of transduced cells 2 days after transduction = 20-40%). Transduced (sgRNA+) cells were selected with 2 doses of puromycin (1 µg/mL) up to 80-95% sgRNA+ in the population over the course of 5 days. Before the initiation of compound treatment, T0 samples were harvested with a minimum 1000-fold library coverage (approximately 100 million cells). The remaining cells were then divided into 5 treatment arms (DMSO, 1 nM sapanisertib, 1 nM rapamycin, 1 nM sapanisertib + rapamycin, and 1 nM RapaLink-1) with 2 biological replicates each. Cells were monitored for population doublings daily, and dilutions were made using complete media supplemented with the indicated compounds to maintain continuous selective pressure. Cells were cultured at a minimum 500-fold library coverage (approximately 50 million cells) over 10 days, after which T10 samples

were harvested with a minimum 1000-fold library coverage (approximately 100 million cells). Genomic DNA (gDNA) was extracted from T0 and T10 samples using NucleoSpin Blood XL (Macherey-Nagel). sgRNA protospacers were amplified directly from gDNA and processed for sequencing on an Illumina HiSeq 4000 as described previously (*69*).

Screen processing

Sequencing data from CRISPRi and CRISPRa screens were aligned to the hCRISPRi-v2 or hCRISPRa-v2 library respectively, counted, and quantified using the Python 2.7-based ScreenProcessing pipeline [https://github.com/mhorlbeck/ScreenProcessing (*29*)]. Phenotypes and Mann-Whitney *P* values were determined as described previously (*28*, *29*), although data detailed herein are not normalized to total population doublings. Additional analysis and plotting were performed in Prism 9 (GraphPad Software).

Large-scale chemogenomic profiling

High-throughput cell viability determination

High-throughput drug screening and sensitivity modeling (curve fitting and IC_{50} estimation) was performed essentially as described previously (*37*). Cells were grown in RPMI or DMEM/F12 medium supplemented with 5% FBS and penicillin/streptomycin, and maintained at 37°C in a humidified atmosphere at 5% CO_2. Cell lines were propagated in these two media in order to minimize the potential effect of varying the media on sensitivity to therapeutic compounds in our assay, and to facilitate high-throughput screening. To exclude cross-contaminated or synonymous lines, a panel of 92 SNPs was profiled for each cell line (Sequenom, San Diego, CA) and a pair-wise comparison score calculated. In addition, short tandem repeat (STR) analysis (AmpFlSTR Identifiler, Applied Biosystems, Carlsbad, CA) was performed and matched to an existing STR profile generated by the providing repository. Briefly, cells were seeded in 384 well plates at variable density to insure optimal proliferation during the assay. Drugs were added to the cells the day after seeding for adherent cell lines and the day of seeding for suspension cell lines. For tumor subtypes containing both adherent and suspension cells, all lines where drugged the same day (small cell lung cancer cell lines for example were all drugged the day after seeding). A series of nine doses was used with a 2-fold dilution factor for a total concentration range of 256 fold. Viability was determined using resazurin after 5 days of drug exposure, and data from treated wells were normalized to that of untreated wells.

Correlation analysis between drug sensitivity and basal gene expression

Dose-dependent growth inhibition of 935 cancer cell lines by RapaLink-1 and sapanisertib was determined as described above. Growth inhibition of 745 cell lines by rapamycin was obtained from the Genomics of Drug Sensitivity in Cancer database (GDSC2 release 8.3, accessed Oct. 4, 2020) (*37*, *39*). Gene expression data, reported as $\log_2$ transformed transcripts per million with a pseudocount of 1, was obtained from the DepMap (21Q4 release) (*70*). Spearman correlation coefficient between transcript level and area under the dose-response curve was calculated for each transcript using all cell lines present in both datasets (659 for RapaLink-1 and sapanisertib, 555 for rapamycin). Analysis and calculations (https://github.com/dwassarman/cellpanelr version 0.0.0.9001) were performed in R using tidyverse (*71*) and DepMap (*70*) packages and plotted in Prism 9 (GraphPad Software).

Cloning of single sgRNA expression vectors

sgRNA protospacers targeting *FKBP12* (also known as *FKBP1A*), *IFITM1*, *IFITM2*, *IFITM3*, and a negative control (NegCtrl) sequence were individually cloned into pCRISPRia-v2 (Addgene 84832) as described previously (*29*). Protospacer sequences are listed in table S2. First, complementary synthetic oligonucleotide pairs (Integrated DNA Technologies) were designed containing protospacer sequences and flanking BstXI and BlpI restriction sites. Complementary oligonucleotides were mixed (2 μM each) in Nuclease-Free Duplex Buffer (Integrated DNA Technologies) and annealed by heating at 95 °C for 5 min, followed by gradual cooling to room temperature on the benchtop for 30 min. These duplexes were then ligated with BstXI, BlpI (New England Biolabs) doubly digested pCRISPRia-v2 (Addgene 84832) using T4 DNA Ligase (New England Biolabs). Standard transformation and preparation protocols were used to isolate individual vectors, which were sequence verified by Sanger sequencing (Quintara Biosciences).

Cloning of triple sgRNA expression vectors
sgRNA protospacers targeting *IFITM1*, *IFITM2*, and *IFITM3* or three negative control protospacers were cloned into pCRISPRia-v2 (Addgene 84832) using a two-step procedure as described previously (*40*) to generate a vector expressing three sgRNAs. Protospacer sequences and their corresponding vectors are listed in table S2. First, complementary oligonucleotides were mixed (2 μM each) in Nuclease-Free Duplex Buffer (Integrated DNA Technologies) and annealed by heating at 95 °C for 5 min, followed by gradual cooling to room temperature on the benchtop for 30 min. These duplexes were then ligated with BstXI, BlpI (New England Biolabs) doubly digested intermediate backbones pMJ114 (Addgene 85995), pMJ179 (Addgene 85996), or pMJ117 (Addgene 85997) using T4 DNA Ligase (New England Biolabs). Standard transformation and preparation protocols were used to isolate individual vectors, which were sequence verified by Sanger sequencing (Quintara Biosciences). Then, single cassettes from each of the three intermediates vectors were polymerase chain reaction (PCR) amplified, gel purified, and inserted into XbaI, XhoI (New England Biolabs) doubly digested pCRISPRia-v2 (Addgene 84832) using NEBuilder HiFi DNA Assembly Master Mix (New England Biolabs) in a single four-piece Gibson assembly step. Standard transformation and preparation protocols were used to isolate individual vectors, which were sequence verified by Sanger sequencing (Quintara Biosciences).

Stable cell line generation
K562 CRISPRi or CRISPRa cells (200,000 cells in 1 mL per well) were seeded into 24-well plates and treated with lentivirus containing sgRNA expression vectors [marked with a puromycin resistance cassette and blue fluorescent protein (BFP)] in the presence of polybrene (8 μg/mL). 2 days after transduction, cells were selected for sgRNA+ populations with 3 doses of puromycin (2 μg/mL) over the course of 6 days. These cells could be stored under cryogenic conditions and were used for additional experiments described herein. The stability of cells were monitored by flow cytometry on an Attune NxT (Thermo Fisher Scientific), maintaining fluorescent marker expressing populations ≥ 90%.

Individual evaluation of sgRNA phenotypes
Cells were transduced as described herein. 5 days after transduction, cells were divided into 5 treatment conditions (DMSO, 1 nM sapanisertib, 1 nM rapamycin, 1 nM sapanisertib + rapamycin, and 1 nM RapaLink-1). Cells were monitored for the percentage of sgRNA+ (BFP+)

populations daily by flow cytometry, and dilutions were made using complete media supplemented with the indicated compounds to maintain continuous selective pressure. Increased relative sgRNA+ percentage over time corresponded to a resistance chemical-genetic interaction while decreased relative sgRNA+ percentage corresponded to a sensitizing chemical-genetic interaction.

Immunoblotting
Cells (500,000 cells in 2 mL per well) were seeded into 6-well plates and incubated at 37 °C overnight. Following treatment with compounds at the concentrations and times indicated, cells were placed over ice, transferred to 2 mL microcentrifuge tubes, and pelleted at 500*g*, 4 °C. The pelleted cells were washed twice with ice-cold phosphate-buffered saline (PBS) and stored at -80 °C. Pellets were disrupted using lysis buffer [100 mM Hepes (pH 7.5), 150 mM NaCl, and 0.1% NP-40] supplemented with cOmplete Protease Inhibitor Cocktail Tablets (Roche) and PhosSTOP (Roche), and protein concentrations of clarified lysates were determined by protein BCA assay (Thermo Fisher Scientific). Proteins were separated by polyacrylamide gel electrophoresis (PAGE), transferred to 0.2 µm pore size nitrocellulose membranes (Bio-Rad) and blocked using blocking buffer [5% bovine serum albumin (Millipore) in Tris-buffered saline, 0.1% Tween 20 (TBST) supplemented with 0.02% NaN_3]. Membranes were probed with primary antibodies against FKBP12 (ab58072) from Abcam and p-ABL1^{Y245} (2861), p-4EBP1$^{T37/46}$ (2855), p-AKT^{S473} (4060), p-S6$^{S235/236}$ (4858), IFITM1 (13126), IFITM2 (13530), IFITM3 (59212), p-STAT5^{Y694} (4322), and Tubulin (3873) from Cell Signaling Technology diluted (1:1000) in blocking buffer. After primary antibody incubation, membranes were treated with IRDye secondary antibodies (LI-COR Biosciences) according to manufacturer's recommendations and scanned on an Odyssey Imaging System (LI-COR Biosciences). Immunoblot scans were processed using ImageStudioLite 5.2.5 (LI-COR).

Cell viability assays
K562 CRISPRi or CRISPRa cells stably expressing sgRNAs (1,000 cells in 90 µL per well) were seeded into white opaque 96-well plates and incubated at 37 °C overnight. Cells were treated with the indicated concentrations of compound in 9-point 3-fold dilution series (100 µL final volume per well) and incubated at 37 °C for 72 h. Cell viability was assessed by CellTiter-Glo (CTG) Luminescent Cell Viability Assay (Promega). Cells were equilibrated to room temperature before the addition of diluted (1:4 CTG reagent:PBS) CTG reagent (100 µL per well). Plates were agitated on an orbital shaker and luminescence signal was measured on a SpectraMax M5 (Molecular Devices) or Spark (Tecan) plate reader. Repeated measurements of luminescence were performed as technical replicates to determine incubation times optimal for signal-to-noise. Luminescence measurements were normalized to DMSO-treated values to determine relative cell viability. Combined CRISPRi/a cellular potency modulation for RapaLink-1, DasatiLink-1, and BisRoc-1 was calculated by the following: [(CRISPRi targeting sgRNA IC_{50}) ÷ (CRISPRi negative control sgRNA IC_{50})] × [(CRISPRa negative control sgRNA IC_{50}) ÷ (CRISPRa targeting sgRNA IC_{50})]. For experiments in which cells were monitored for population doublings over multiple days, K562 CRISPRi cells stably expressing sgRNAs were seeded into 12-well plates (1,000 cells in 2 mL per well) and incubated at 37 °C over the course of the experiment. Every 2 days, 200 µL of cell suspension was removed for cell counting by flow cytometry on an Attune NxT (Thermo Fisher Scientific) using a FSC × SSC gate.

Internally normalized cellular fluorescence uptake assay

K562 CRISPRi or CRISPRa cells stably expressing sgRNAs marked with BFP mixed at a 1:1 ratio with non-transduced (sgRNA-) cells (20,000 cells in 180 µL per well) were seeded into 96-well round bottom plates and incubated at 37 °C overnight. Cells were treated with fluorescent compounds at the concentrations indicated (200 µL final volume per well) and incubated at 37 °C for 24 h. Cells were pelleted at 500*g*, washed twice with ice-cold PBS supplemented with 1% bovine serum albumin (Millipore) and 0.1% NaN_3, and resuspended in the same before assessment by flow cytometry on an Attune NxT (Thermo Fisher Scientific). TAMRA fluorescence (YL-H: 561 nm excitation laser, 585/16 emission filter) and BFP fluorescence (VL1-H: 405 nm excitation laser, 440/50 emission filter) was measured for cells within each well. Relative cellular uptake was determined by dividing the median TAMRA fluorescence intensity of BFP+ populations by that of BFP- populations (Fig. 2B). Relative cellular uptake < 1 indicates decreased uptake resulting from the genetic perturbation and > 1 indicates increased uptake.

Confocal microscopy

RPE1 CRISPRi (dCas9-KRAB) cells (a generous gift from Ron Vale) stably expressing sgRNAs were seeded into 35 mm glass bottom dishes (MatTek #P35G-1.5-14-C) and treated with 100 nM RapaTAMRA-PEG8 and 5 µM LysoTracker Green DND-26 (Invitrogen #L7526) for 24 hours. Prior to imaging, cells were washed three times with PBS then imaged in HEPES-buffered and phenol red-free DMEM (Gibco #21063029) supplemented with 10% FBS. Live-cell microscopy was carried out on an incubated stage using a Nikon Ti microscope equipped with a Yokogawa CSU-22 spinning disk confocal and Photometrics Evolve Delta EMCCD camera using an argon laser line at 488 nm (used to excite Oregon Green 488 or EGFP), a HeNe laser line at 543 nm (used to excite Texas RedTM or cresyl violet) and a HeNe laser line at 633 nm (used to excite Alexa 647). Band pass (505–530 nm) and long pass (560 nm) filters were used to separate emission wavelengths of Oregon Green 488 and Texas RedTM respectively. Laser power was attenuated to 2% of maximum to minimize photobleaching and phototoxicity. The detector pinholes were set to give a 0.7-1.4 µm optical slice. Pixel dwell times varied from 1 µs to 1.58 µs using multitracking (line switching) with a line average of 4. Image acquisition was performed in NIS-elements software using a Plan Apo VC 60X/1.4 oil-immersion DIC N2 objective lens. Images were acquired from randomly selected fields of view and then exported to Inkscape for display or ImageJ for quantification. For quantification, cells were segmented manually and thresholded in the LysoTracker channel to generate a lysosome mask. Pixel intensity was measured within the cell as a whole, within the lysosome mask (endolysosomal) or within the cell as whole excluding the mask (intracellular). Quantification was performed on > 30 cells per condition from 3 distinct biological replicates.

Protein expression and purification

Human ABL1 kinase domain (KD) encompassing residues 229-512 (isoform IA numbering) was cloned, expressed in *Escherichia coli* (*E. coli*), and purified as described previously (*72*). ABL1 KD containing a tobacco etch virus (TEV) protease-cleavable N-terminal hexahistidine tag (MKSSHHHHHHHHHHENLYFQSNA) was transformed into BL21(DE3) *E. coli* cells carrying a plasmid containing YopH phosphatase (pCDRF-Duet, streptomycin resistant) and a plasmid expressing GroEL and Trigger factor (pACYC-Duet, chloramphenicol resistant). ^{15}N labeled ABL1 KD samples were produced in M9 minimal media containing 1 g/L $^{15}NH_4Cl$ as the sole

nitrogen source. Cells were grown at 37 °C to OD_{600} ~0.6–0.8. At OD_{600} ~0.6–0.8 cells were cooled to 16 °C for an hour, then expression was induced with 1 mM isopropyl-β-D-thiogalactoside (IPTG) and allowed to continue overnight (16-20 h). Proteins were purified with a 5 mL HisTrap HP (GE Healthcare) Ni affinity column (NiA buffer: 20 mM Tris pH 8.0, 500 mM NaCl, 5% glycerol; NiB buffer: 20 mM Tris pH 8.0, 500 mM NaCl, 5% glycerol, 500 mM imidazole), dialyzed overnight with TEV protease in 20 mM Tris pH 8.0, 100 mM NaCl, 1 mM DTT, 5% glycerol, and then purified with a 5 mL HiTrap anion exchange column (QA Buffer: 20 mM Tris pH 8.0, 1 mM TCEP, 5% glycerol; QB Buffer: 20 mM Tris pH 8.0, 1 M NaCl, 1 mM TCEP, 5% glycerol). Protein concentration was determined by absorbance measurement using a calculated extinction coefficient of 62590 $M^{-1}cm^{-1}$ (ProtParam) (*73*). Purified samples were concentrated to 300 µM by ultrafiltration (molecular weight cut-off 10 kDa) and buffer exchanged into 50 mM sodium potassium phosphate pH 6.5, 50 mM NaCl, 5 mM DTT. Samples were snap frozen in liquid nitrogen and stored at -80 °C.

NMR experiments

Dasatinib, asciminib, and their combination were added in five-fold molar excess to saturate binding sites during buffer exchange. For DasatiLink-1, the protein was diluted to ~60 µM in 2500 µL and 25 µL of 5 mM bitopic ligand was added on ice to minimize solute precipitation. The process was repeated until the bitopic ligand reached 3-fold excess of the protein concentration. DMSO was maintained at 5% for all NMR samples. Samples were concentrated to a final protein concentration of 300 µM. 10% D_2O was added to NMR samples for signal locking. All ^1H-^{15}N heteronuclear NMR experiments were acquired at 30 °C with 64 scans on a Bruker Avance III HD spectrometer operating at a ^1H frequency of 850 MHz equipped with a cryogenic probe. A standard Bruker pulse sequence for ^1H-^{15}N TROSY-HSQC (trosyf3gpphsi19.2), was used. Backbone assignments for ABL1 KD used to interpret spectra described herein were obtained from the Biological Magnetic Resonance Data Bank (Entry ID: 15488) (*74*). Sample stability prior and post NMR experiments was assessed by acquiring 1-dimensional ^1H spectra to assess signal strength, sample concentration, and folding status.

ATP-site kinase pulldown

ATP-site competition binding assay (*Kd*ELECT) was performed by Eurofins DiscoverX as described previously (*59*). Compounds were assessed in 11-point 3-fold dilution series and compound mixtures were analogously diluted from a DMSO stock containing the 2 compounds at the ratio indicated. Pulldown measurements of DNA-tagged kinase by quantitative polymerase chain reaction (qPCR) were normalized to DMSO-treated values to determine relative ATP-site pulldown. A 4-parameter nonlinear regression model was fit to the data using Prism 9 (GraphPad Software) with the top parameter constrained to 100%. An outlier point corresponding to 152% relative ATP-site pulldown at 15.2 pM Dasatinib + Asciminib (1:100) was excluded from analysis and plotting in fig. S8C due to high likelihood of technical error associated with the measurement.

Live cell kinase occupancy profiling

Compound treatment and preparation of cell lysates for proteomics analysis

K562 CRISPRi cells (1×10^6/mL) were maintained in RPMI medium (Gibco) supplemented with 10% (v/v) fetal bovine serum (FBS) (Axenia BioLogix), penicillin (100 U/mL, Gibco), and streptomycin (100 µg/mL, Gibco). Cells were pretreated with DMSO, dasatinib + asciminib (10

nM, 100 nM, or 1 μM), or DasatiLink-1 (10 nM, 100 nM, or 1 μM) at 37 °C for 4 h, followed by treatment with XO44 (2 mM) at 37 °C for another 30 min. Each sample was prepared in triplicate. Cell pellets were collected by centrifugation at 500*g*, 4 °C and lysed in 100 mM HEPES pH 7.5, 150 mM NaCl, 0.1% NP-40, 1 mM PMSF, and 1× cOmplete EDTA-free protease inhibitor cocktail (Sigma-Aldrich #11873580001). Lysates were cleared by centrifugation (16,000*g*, 4 °C, 30 min). Protein concentration was determined by protein BCA assay (Thermo Fisher #23225). Cell lysates were normalized to 5 mg/mL with lysis buffer for subsequent pulldown-MS analysis.

Pulldown of XO44-modified proteins and on-bead digestion
Cell lysates (5 mg/mL, 1.2 mL) were incubated with 40 mL of settled streptavidin agarose beads (Thermo Fisher Scientific #20353) at 4 °C overnight to remove endogenous biotinylated proteins. Beads were removed by filtration (Pall #4650). The filtrate (1 mL) was reacted with 191 mL of click chemistry cocktail, resulting in a final concentration of 1% SDS, 100 mM DMTP biotin picolyl azide, 1 mM TCEP, 100 mM TBTA (from a 2 mM stock prepared in 1:4 DMSO:t-butyl alcohol), and 1 mM $CuSO_4$. After incubation at room temperature for 90 min, proteins were precipitated by adding 10 mL of prechilled acetone and incubating overnight at –20 °C. The precipitated proteins were pelleted by centrifugation (3500*g*, 4 °C, 30 min), resuspended in cold MeOH and re-pelleted. The pellet was solubilized in 1% SDS in PBS, and then diluted to a final detergent concentration of 0.4% SDS, 0.6% NP40 in PBS before desalting on a NAP-10 column (Cytiva #17-0854-02). The column eluate was incubated with 40 mL of settled high-capacity neutravidin garose beads (Thermo Fisher Scientific #29204) at 4 °C overnight. The beads were then washed with 1% NP-40, 0.1% SDS in PBS (3 x 10 min, RT), freshly prepared 6 M urea in PBS (3 x 30 min, 4 °C) and PBS (3 x 10 min, RT). Disulfide reduction was performed with 5 mM DTT in 6M urea, PBS at 56 °C for 30 min, followed by alkylation with 20 mM iodoacetamide at room temperature for 15 min in the dark. On-bead digestion was performed in digestion buffer (2 M urea, 1 mM $CaCl_2$, PBS) by adding 1 mg sequencing grade trypsin (Promega #V5113) to each sample, and incubating overnight at 37 °C. Tryptic digests were collected by filtration. Peptide concentrations were determined by peptide BCA assay (Thermo Fisher Scientific #23275). An equal amount of peptides were removed from each sample and dried by Speedvac.

TMT labeling of tryptic peptides
TMT labeling was performed with the TMT10plex kit (Thermo Fisher Scientific #SK257743) according to manufacturer's recommendations with minor modifications. Briefly, peptides (25 μg) were reconstituted in 50 μL of 30% MeCN in 200 mM HEPES buffer pH 8.5. TMT reagents were reconstituted in 40 μL of MeCN per vial, and 6 μL of this solution was incubated with each sample for 1 h at RT. Reactions were quenched by adding 9 μL of 5% hydroxylamine and incubated at RT for 15 min, followed by adding 50 μL of 1% TFA to acidify the solution. TMT-labeled samples were pooled and concentrated by Speedvac to remove MeCN, and desalted using C18 OMIX Tips (Agilent #A57003100). Peptides were eluted with 50% MeCN, 0.1% TFA, and dried by Speedvac.

LC-MS/MS analysis
TMT labeled tryptic peptides were reconstituted in 5% MeCN, 0.1% TFA in water, and analyzed on a Orbitrap Eclipse Tribrid Mass Spectrometer (Thermo Fisher Scientific) connected to an

UltiMate 3000 RSLCnano system with 0.1% FA as buffer A and 95% MeCN, 0.1% FA as buffer B. Peptides were separated on an EASY-Spray 3 μm, 75 μm × 15 cm C18 column (Thermo Fisher Scientific #ES800) with the following LC settings: 0.3 mL/min flow rate, sample loading at 5% B for 20 min, then 5 to 7.4% B over 5 min, 7.4 to 50% B over 115 min, 50% to 95% B over 10 min and finally 95% B for 10 min. Data were acquired in a data-dependent mode. MS1 scans were acquired at a resolution of 120,000 with an AGC of 4e5, m/z scan range of 400-1600, a maximum ion injection time of 50 ms, a charge state of 2-6, and a 60 s dynamic exclusion time. MS2 spectra were acquired via collision-induced dissociation (CID) at a collision energy of 35%, in the ion trap with an automatic gain control (AGC) of 1e4, isolation width of 0.7 m/z and an auto maximum ion injection time. For real time search, MS2 spectra were searched against human reviewed Swiss-Prot database (accessed Sept. 16, 2020) with the digestion enzyme set to trypsin. Methionine oxidation was set as a variable modification, while carbamidomethylation of cysteine and TMT modification were set as constant modifications. For MS3 acquisition, a synchronous precursor selection (SPS) of 10 fragments was acquired in the orbitrap for a maximum ion injection time of 105 ms with an AGC of 2.5e5. MS3 spectra were collected at a resolution of 60,000 with higher-energy C-trap dissociation (HCD) collision energy of 55%.

Protein identification and TMT quantification.
Raw files were analyzed with Thermo Scientific Proteome Discoverer (2.4) software against the human reviewed Swiss-Prot database (accessed Sept. 16, 2020). Trypsin was selected as the digestion enzyme with a maximum of 2 missed cleavages and a minimum peptide length of 6. Cysteine carbamidomethylation and TMT-6plex on K and peptide N-terminus were set as fixed modifications, while methionine oxidation and acetylation of protein N-terminus were set as variable modifications. Precursor tolerance was set to 10 ppm, and fragment tolerance was set to 0.6 Da. Peptide-spectrum match (PSM) and protein false discovery rate (FDR) were set to 1% and 5%, respectively. Reporter ion intensities were adjusted to correct for impurities during synthesis of different TMT reagents according to the manufacturer's recommendations. For quantification, PSMs with an average reporter signal-to-noise threshold (< 9) and synchronous precursor selection (SPS) mass matches threshold (< 75%) were removed from final dataset. Quantified PSMs were summarized to their matched proteins. Median protein intensities for each TMT channel were used to normalize protein intensities across all channels. Normalized treatment intensities for each replicate were divided by DMSO values, log_2 transformed, and averaged for calculation of the mean log_2 fold change for each condition (e.g. dasatinib + asciminib or DasatiLink-1). Contaminant keratin (KRT) proteins were excluded from plots.

Chemical-genetic interaction mapping
Cells treated with compounds were evaluated for viability as described herein. For K562 CRISPRi cells JQ1 (10 μM), asciminib (100 nM), rocaglamide (1 μM), HJB97 (10 μM), dasatinib (100 nM), sapanisertib (1 μM), GNF-2 (10 μM), rapamycin (100 nM), GMB-475 (10 μM), BisRoc-3 (1 μM), MZ1 (10 μM), BETd-260 (100 nM), BisRoc-2 (1 μM), dBET6 (1 μM), BisRoc-1 (1 μM), DasatiLink-1 (100 nM), and RapaLink-1 (100 nM) were evaluated using 9-point 3-fold dilution series starting from the highest concentrations indicated. The same top concentrations were used in K562 CRISPRa cells with the exception of HJB97 (1 μM), JQ1 (1 μM), MZ1 (1 μM), and dBET6 (100 nM). Using Prism 9 (GraphPad Software), a 4-parameter nonlinear regression model was fit to the viability data to determine IC_{50} values. IC_{50} values of sgRNA+ cells were normalized to that of non-sgRNA expressing cells to determine

sensitivity/resistance chemical-genetic interactions mapped in Fig. 4B (see also Fig. 1F, Fig. 3D, or fig. S9D for example source data).

Chemical space plotting

Data were drawn from the Protein Kinase Inhibitor Database (PKIDB) (*75*) and the Proteolysis-Targeting Chimera Database (PROTAC-DB) (*76*). 304 kinase inhibitors from the PKIDB (July 18, 2022 release, accessed August 24, 2022) and 3270 PROTACs from the PROTAC-DB (June 17, 2022 release, accessed August 24, 2022) were depicted in Fig. 4C. Molecular weight and topological surface area were plotted based on values associated with compounds in their respective databases. For other linked inhibitors, physicochemical properties were computed as described herein.

Physicochemical property determination

Unless otherwise specified, physicochemical properties of compounds were computed using the Mcule property calculator (*77*) and listed in table S1. Simplified molecular-input line-entry system (SMILES) strings were inputted to https://mcule.com/apps/property-calculator/.

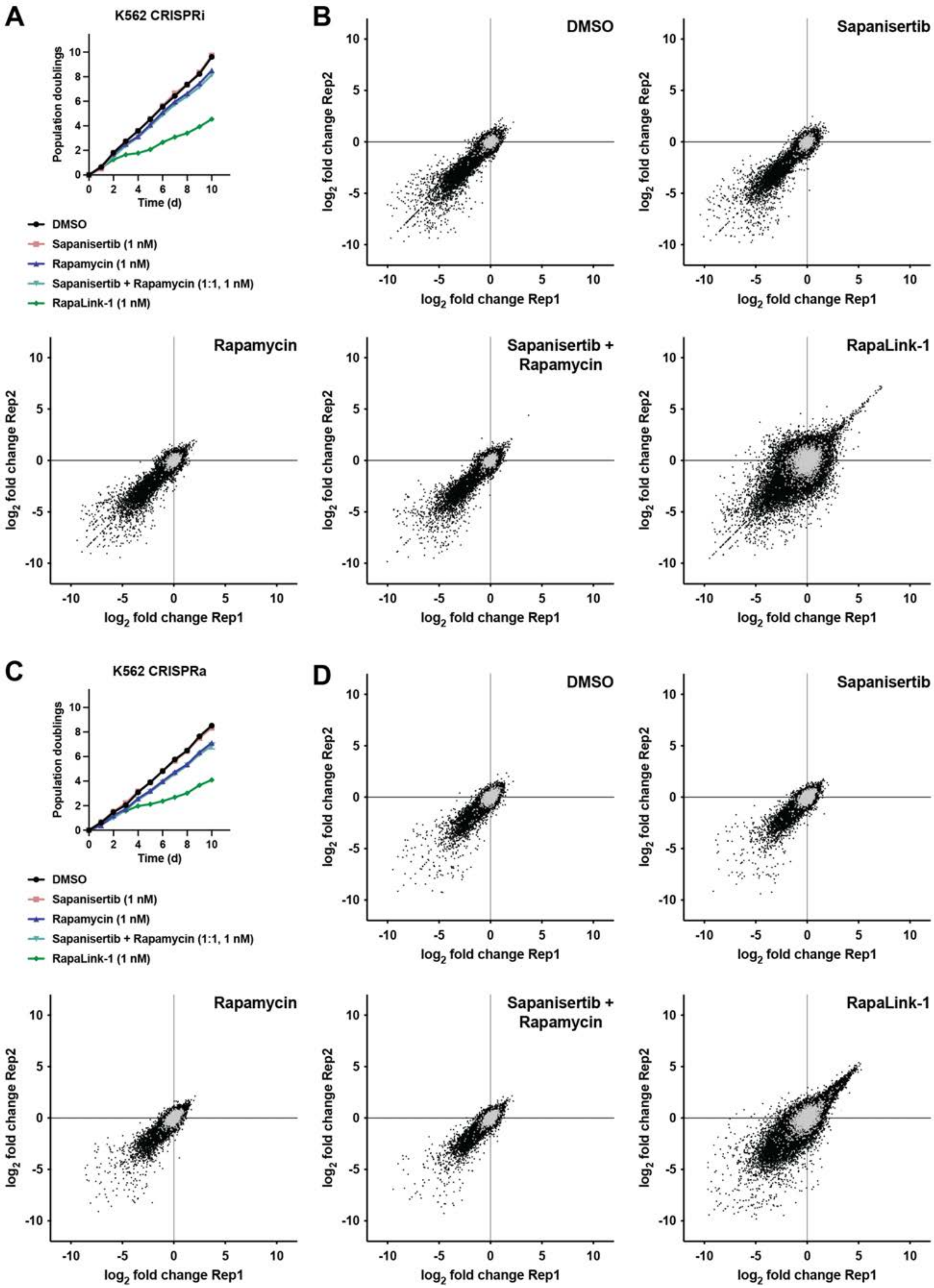
A
K562 CRISPRi
Population doublings
Time (d)
DMSO
Sapanisertib (1 nM)
Rapamycin (1 nM)
Sapanisertib + Rapamycin (1:1, 1 nM)
RapaLink-1 (1 nM)
B
DMSO
Sapanisertib
Rapamycin
Sapanisertib + Rapamycin
RapaLink-1
log2 fold change Rep1
log2 fold change Rep2
C
K562 CRISPRa
D

Fig. S1. CRISPRi/a screening in K562 cells identifies genes that determine cellular response to MTOR inhibitors.

(**A**) Population doublings of K562 CRISPRi cells over the course of functional genomics screens. Arms correspond to continuous inhibitor treatment with the indicated concentrations. Data represent means of two biological replicates; error bars denote SD. (**B**) sgRNA phenotypes derived from growth selections in (A). Targeting sgRNAs (black) and non-targeting sgRNAs (gray) are plotted for two biological replicates. (**C**) As in (A) for K562 CRISPRa cells. (**D**) As in (B) for K562 CRISPRa cells.

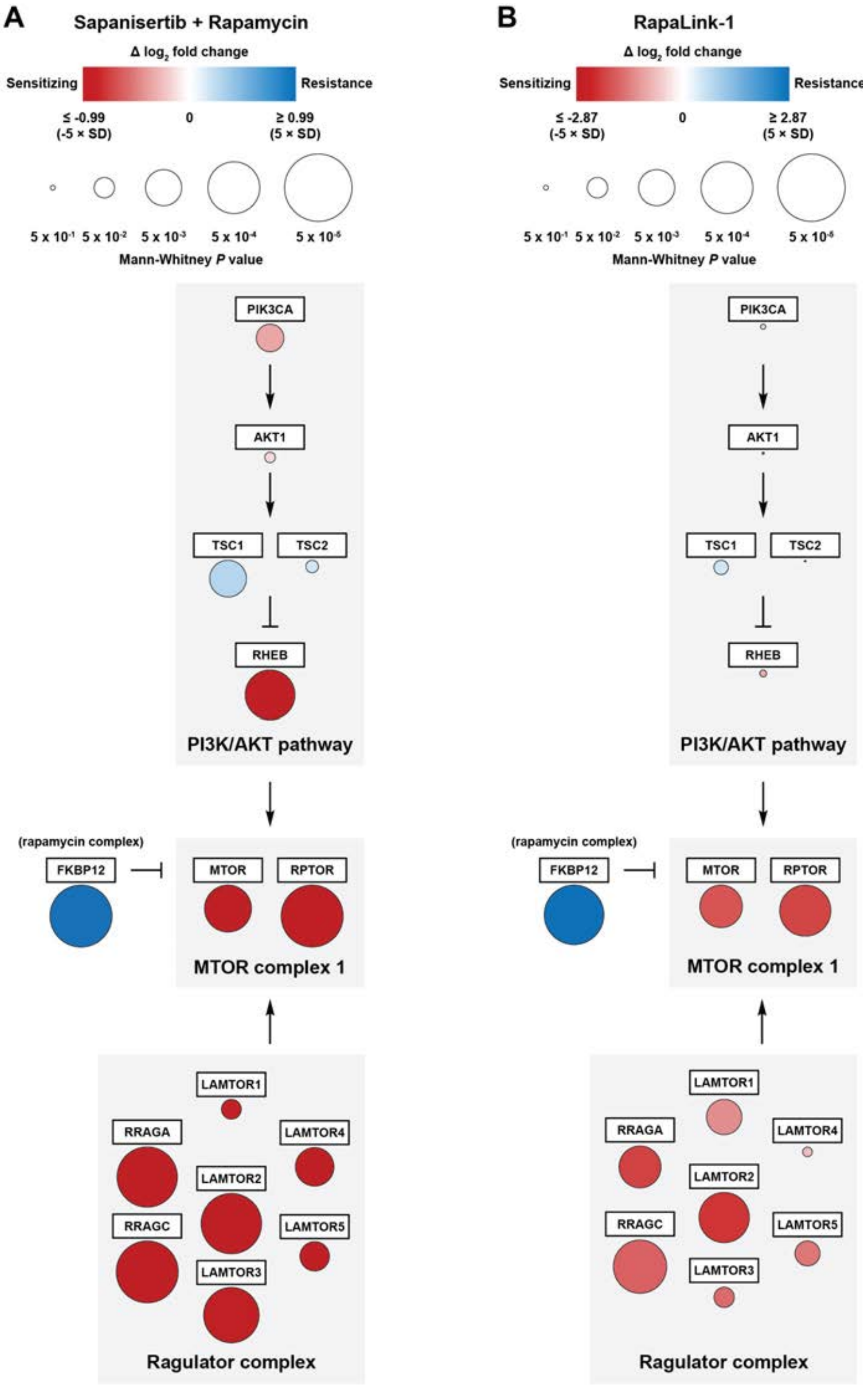

Fig. S2. Established MTOR regulatory mechanisms modulate sensitivity/resistance to MTOR inhibitors.

(**A**) Pathway map of chemical-genetic interactions with a 1:1 mixture of sapanisertib and rapamycin in a genome-scale K562 CRISPRi screen. Color intensities portray phenotype strength and circle diameters represent $-\log_{10}$ Mann-Whitney *P* values. Data represent two biological replicates. (**B**) As in (A) for RapaLink-1.

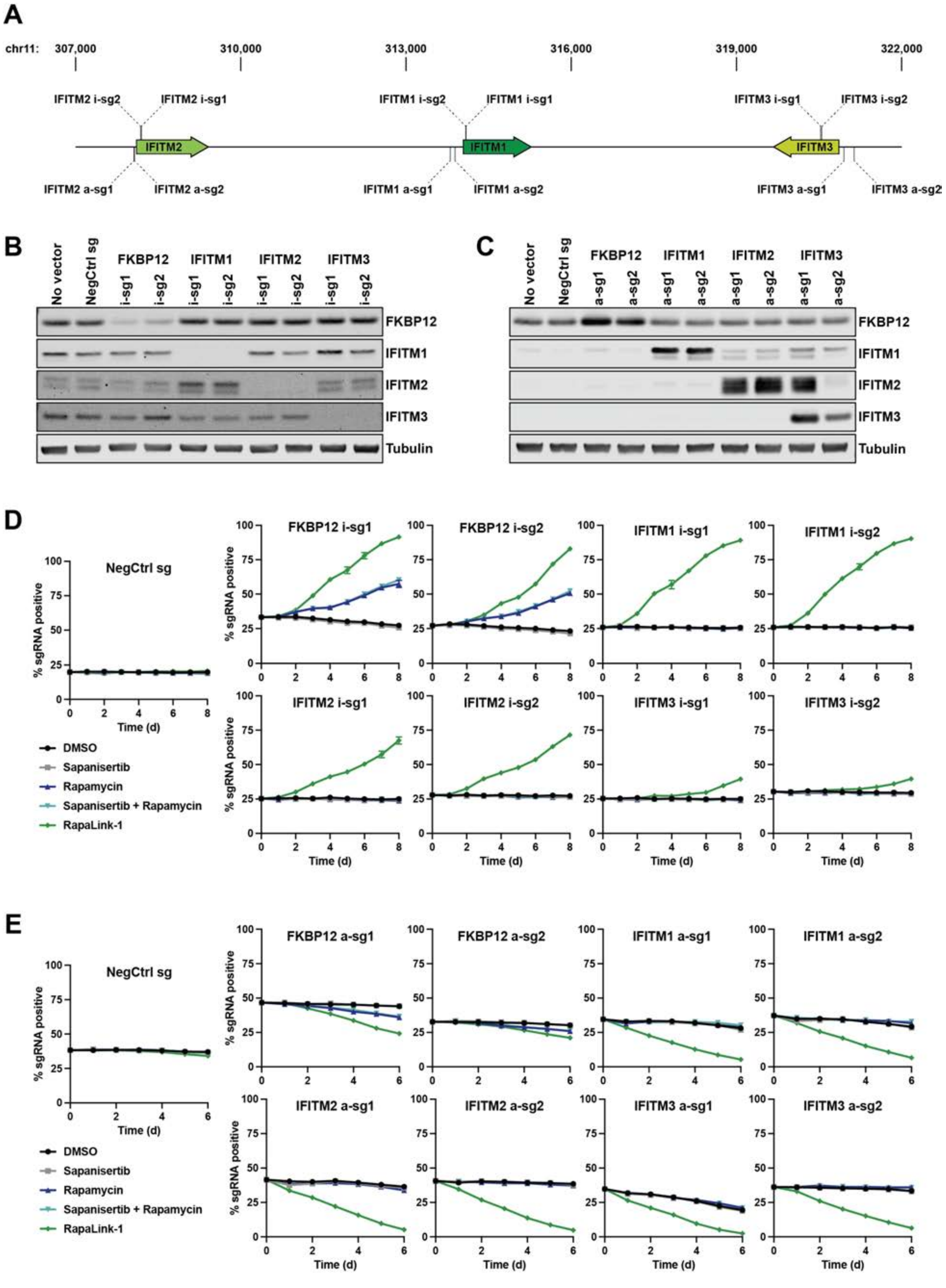

A
chr11: 307,000 310,000 313,000 316,000 319,000 322,000
IFITM2 i-sg2 IFITM2 i-sg1 IFITM1 i-sg2 IFITM1 i-sg1 IFITM3 i-sg1 IFITM3 i-sg2
IFITM2 IFITM1 IFITM3
IFITM2 a-sg1 IFITM2 a-sg2 IFITM1 a-sg1 IFITM1 a-sg2 IFITM3 a-sg1 IFITM3 a-sg2
B
No vector NegCtrl sg FKBP12 IFITM1 IFITM2 IFITM3
i-sg1 i-sg2 i-sg1 i-sg2 i-sg1 i-sg2 i-sg1 i-sg2
FKBP12 IFITM1 IFITM2 IFITM3 Tubulin
C
No vector NegCtrl sg FKBP12 IFITM1 IFITM2 IFITM3
a-sg1 a-sg2 a-sg1 a-sg2 a-sg1 a-sg2 a-sg1 a-sg2
FKBP12 IFITM1 IFITM2 IFITM3 Tubulin
D
NegCtrl sg
FKBP12 i-sg1 FKBP12 i-sg2 IFITM1 i-sg1 IFITM1 i-sg2
IFITM2 i-sg1 IFITM2 i-sg2 IFITM3 i-sg1 IFITM3 i-sg2
% sgRNA positive
Time (d)
DMSO
Sapanisertib
Rapamycin
Sapanisertib + Rapamycin
RapaLink-1
E
NegCtrl sg
FKBP12 a-sg1 FKBP12 a-sg2 IFITM1 a-sg1 IFITM1 a-sg2
IFITM2 a-sg1 IFITM2 a-sg2 IFITM3 a-sg1 IFITM3 a-sg2
% sgRNA positive
Time (d)
DMSO
Sapanisertib
Rapamycin
Sapanisertib + Rapamycin
RapaLink-1

Fig. S3. IFITM protein expression synergizes specifically with RapaLink-1 inhibitory activity in K562 CRISPRi/a cells.

(**A**) Schematic of the human IFITM locus located within chromosome 11 annotated with positions targeted by sgRNAs described herein. (**B**) Immunoblots of K562 CRISPRi cells stably expressing sgRNAs. Cells were collected for assessment 30 days following selection for sgRNA+ cells. Data representative of three biological replicates. (**C**) as in (B) for K562 CRISPRa cells collected for assessment 15 days following selection for sgRNA+ cells. (**D** and **E**) K562 CRISPRi (D) or CRISPRa (E) cells transduced with sgRNAs were grown in the presence or absence of continuous inhibitor treatment (1 nM) as in the corresponding genome-scale screens. Relative populations of transduced (sgRNA+) and non-transduced (sgRNA-) cells were determined by flow cytometry at the indicated times. Data represent means of three biological replicates; error bars denote SD.

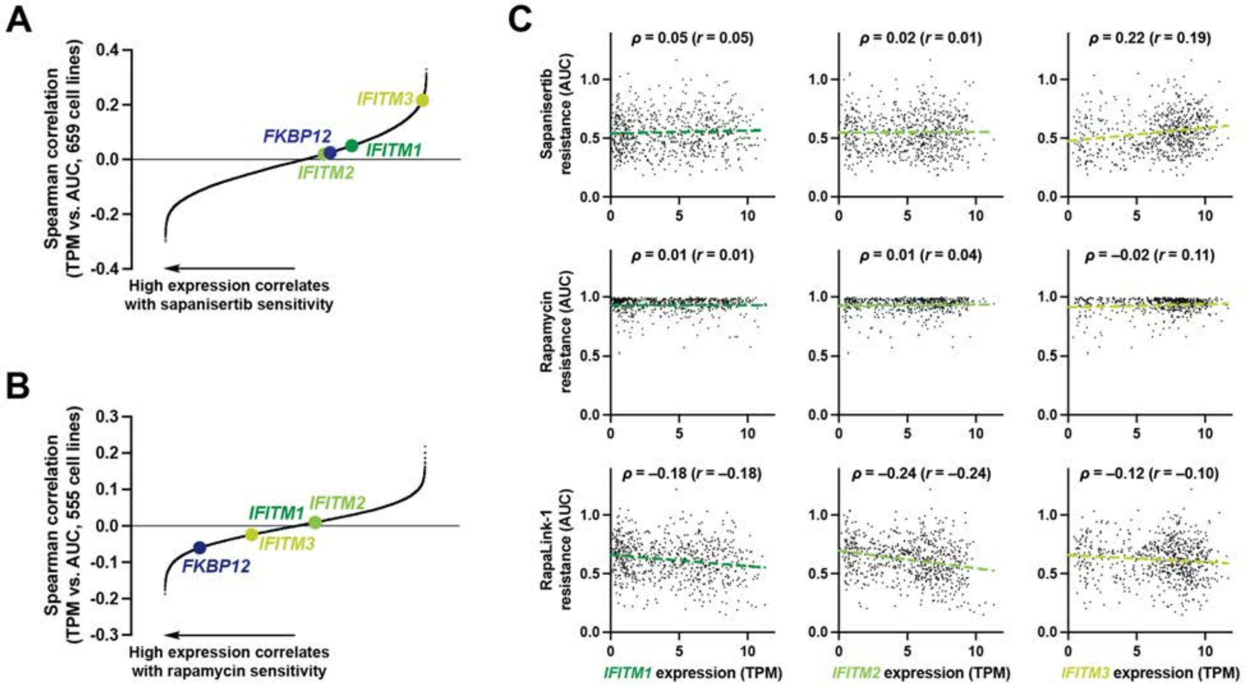

Fig. S4. Basal IFITM protein expression correlates specifically with RapaLink-1 inhibitory activity across diverse cancer cell lines.

(**A** and **B**) Spearman correlation coefficients between sapanisertib (A) or rapamycin (B) sensitivity, as measured by dose-response data, and transcript abundance, as measured by RNA sequencing (see also Fig. 1C). (**C**) Data used to correlate *IFITM1*, *IFITM2*, and *IFITM3* transcript abundance and inhibitor sensitivity in (A and B, and Fig. 1C). Points represent individual cell lines with Spearman correlation coefficients (ρ) indicated for each transcript. Pearson correlation coefficients (r) and linear regressions provided for visualization.

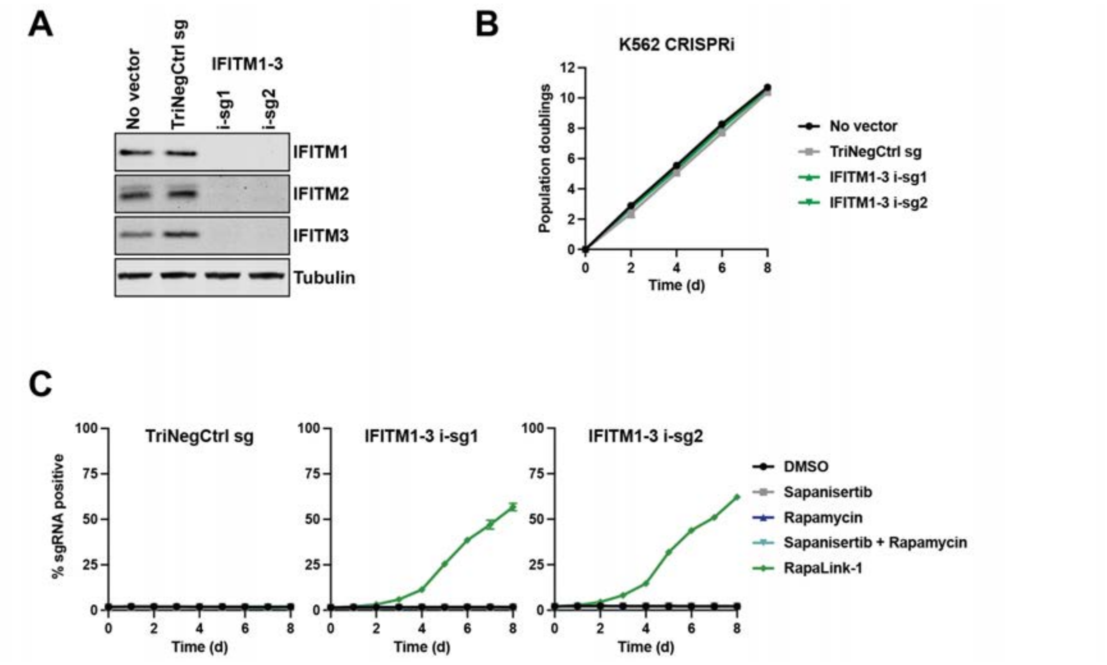

Fig. S5. Simultaneous knockdown of *IFITM1*, *IFITM2*, and *IFITM3* by three sgRNAs confers resistance specifically to RapaLink-1.

(**A**) Immunoblots of K562 CRISPRi cells stably expressing three sgRNAs from a single vector. Cells were collected for assessment 30 days following selection for sgRNA+ cells. Data representative of three biological replicates. (**B**) Population doublings of K562 CRISPRi cells expressing three sgRNAs from a single vector. Data represent means of three biological replicates; error bars denote SD. (**C**) K562 CRISPRi cells transduced with three sgRNAs from a single vector were grown in the presence or absence of continuous inhibitor treatment (1 nM) as in the corresponding genome-scale screens. Relative populations of transduced (sgRNA+) and non-transduced (sgRNA-) cells were determined by flow cytometry at the indicated times. Data represent means of three biological replicates; error bars denote SD.

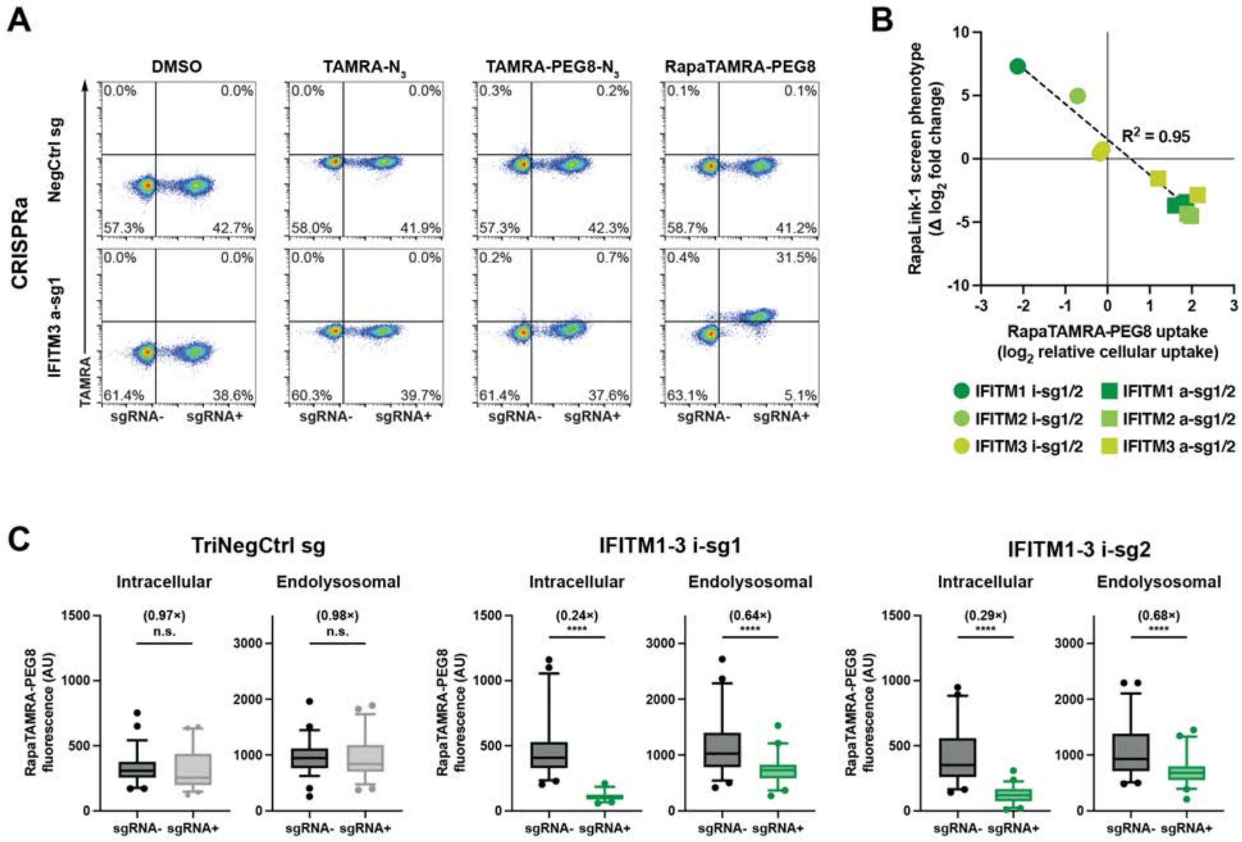

Fig. S6. IFITM proteins promote the intracellular accumulation of linked chemotypes.

(**A**) Measurement of fluorescent molecule uptake in K562 CRISPRa cells expressing sgRNAs (sgRNA+). Cells were incubated with TAMRA-N_3 (10 nM), TAMRA-PEG8-N_3 (1 µM), or RapaTAMRA-PEG8 (1 nM) for 24 h. Data representative of three biological replicates. (**B**) Correlation between relative cellular uptake values for RapaTAMRA-PEG8 in (Fig. 2C) and sensitivity/resistance phenotypes from RapaLink-1 CRISPRi/a screens. (**C**) Box and whisker plots (center, median; box, interquartile range; whiskers, 5-95 percentile; points, outliers) quantifying RapaTAMRA-PEG8 subcellular localization in RPE-1 CRISPRi cells expressing sgRNAs as measured by confocal microscopy (see also Fig. 2D). Median fluorescence data are expressed in arbitrary units (AU). Quantification was performed on > 30 cells per condition across 3 biological replicates. ****P < 0.0001 by an unpaired *t* test. n.s., not significant.

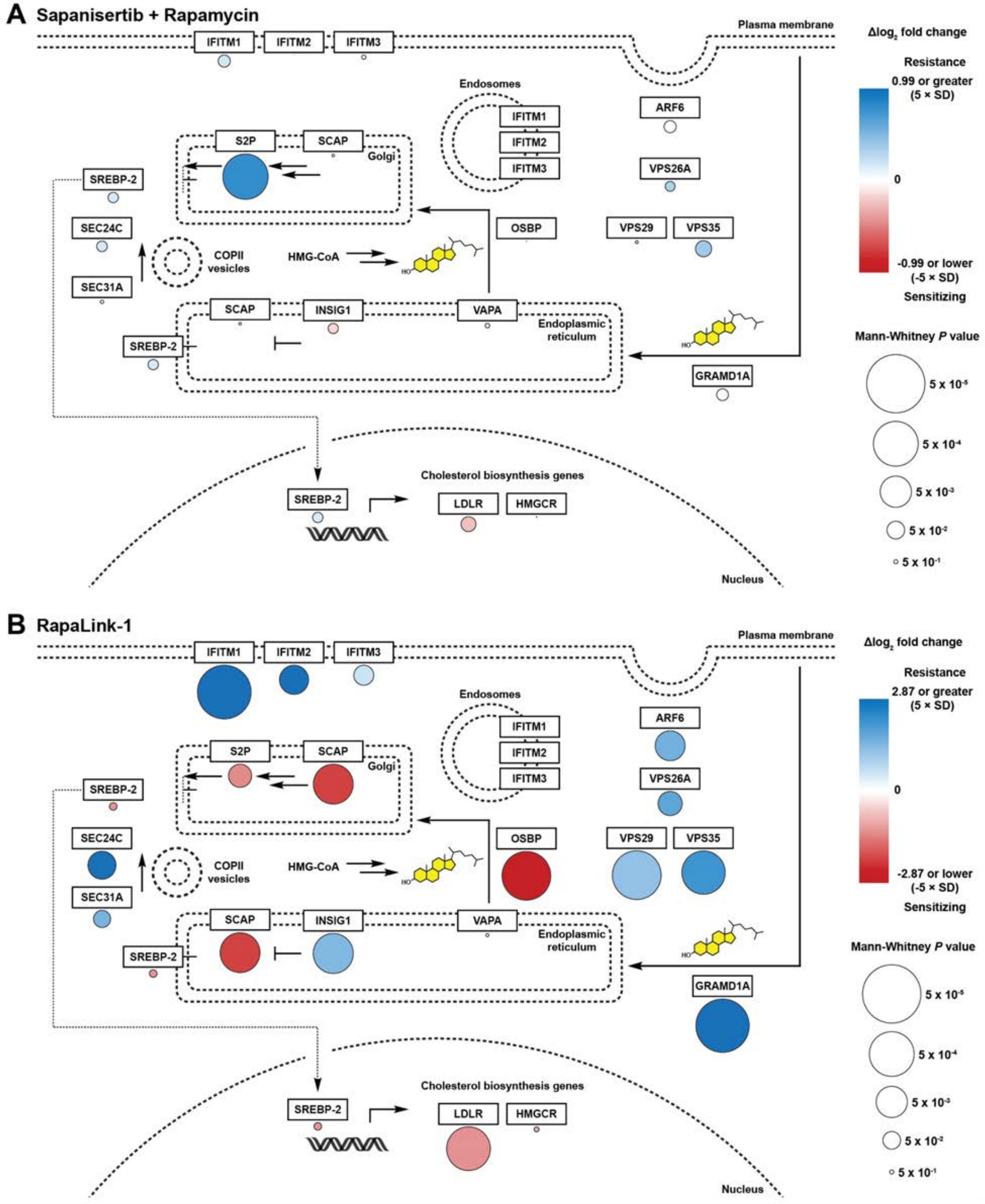

Fig. S7. Endosomal and sterol regulatory pathways specifically modulate RapaLink-1 cellular activity.

(**A**) Pathway map of chemical-genetic interactions with a 1:1 mixture of sapanisertib and rapamycin in a genome-scale K562 CRISPRi screen. Color intensities portray phenotype strength and circle diameters represent $-\log_{10}$ Mann-Whitney *P* values. Data represent two biological replicates. (**B**) As in (A) for RapaLink-1.

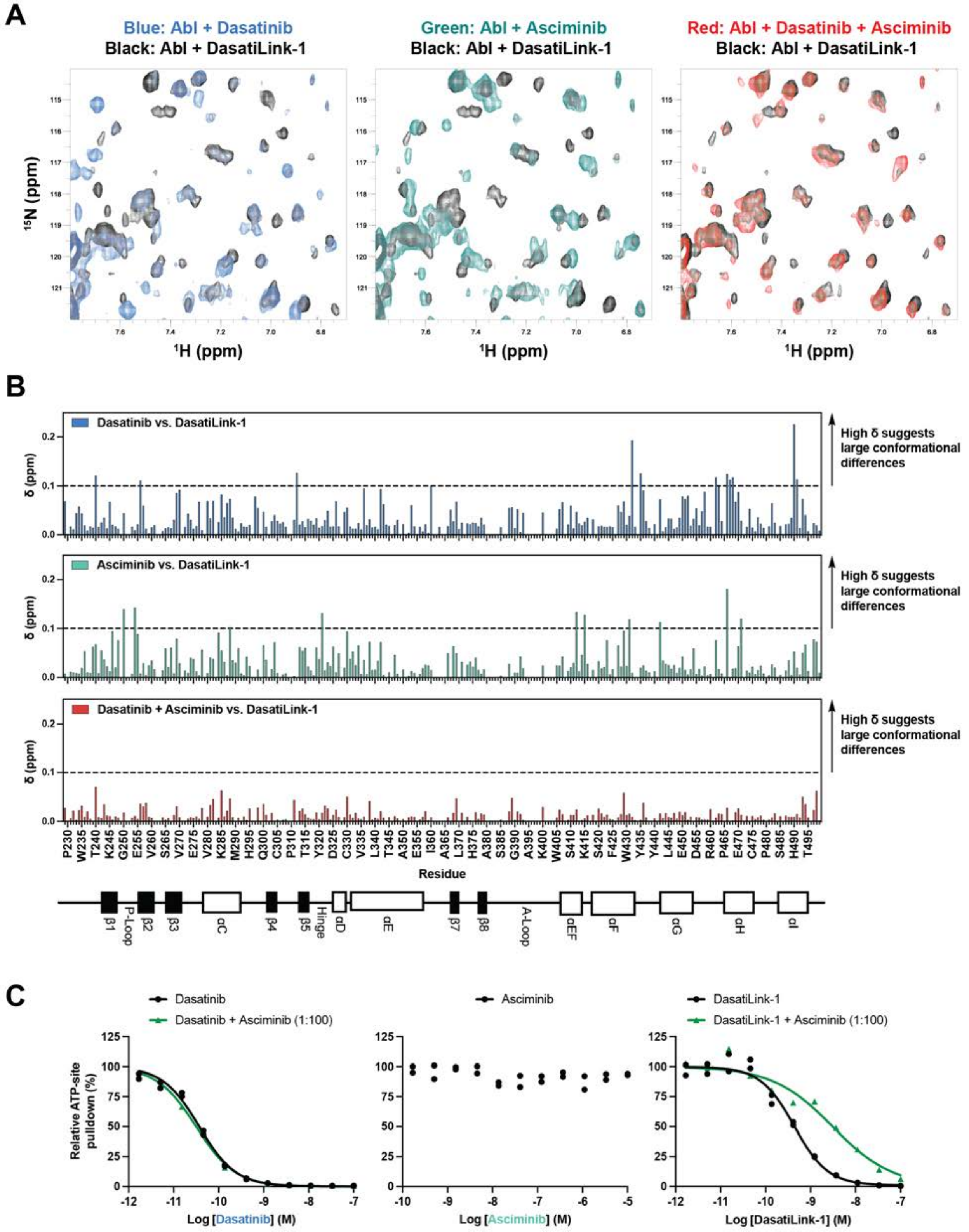

Fig. S8. DasatiLink-1 engages ABL1 kinase through a bitopic mechanism.

(**A**) ^{1}H-^{15}N heteronuclear single quantum coherence (HSQC) spectra of ABL1 kinase domain in the presence of dasatinib (blue), asciminib (green), dasatinib + asciminib (red), and DasatiLink-1 (black). (**B**) Chemical shift differences for assigned residues in ABL1 kinase domain resulting from interactions with different inhibitors as in (A). δ (ppm) > 0.1 indicates a major chemical shift difference. (**C**) ATP-site pulldown of ABL1 kinase domain in the presence of inhibitor with or without addition of 100-fold molar excess asciminib. Data represent two biological replicates.

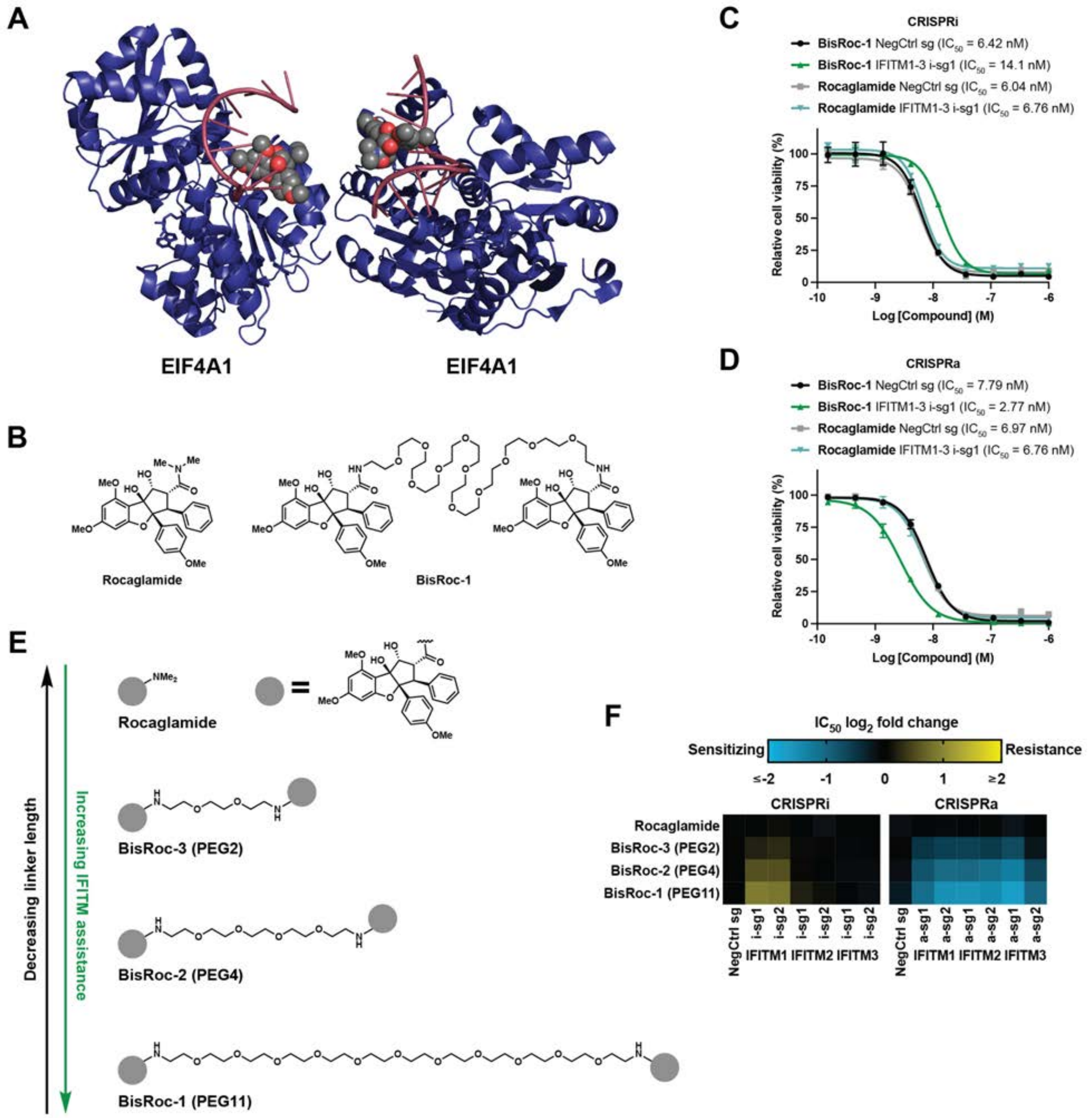

Fig. S9. Longer linker length correlates with greater IFITM assistance in a series of linked rocaglamide analogs.

(**A**) Crystal structure of rocaglamide bound to EIF4A1 and polypurine RNA showing adjacent symmetry mates (PDB, 5ZC9). (**B**) Chemical structures of EIF4A1 inhibitors. (**C** and **D**) Viability of K562 CRISPRi (C) or CRISPRa (D) cells expressing sgRNAs treated with BisRoc-1 or rocaglamide. Data represent means of three biological replicates; error bars denote SD. (**E**) Chemical structures of a BisRoc linker length series. (**F**) Chemical-genetic interaction map of inhibitors with *IFITM1*, *IFTM2*, and *IFITM3*. Potency, as measured by dose-response IC_{50} in a

cell viability assay as in (D), was normalized to that of non-sgRNA-expressing K562 CRISPRi or CRISPRa cells. Data represent means of three biological replicates (excerpted from Fig. 4B).

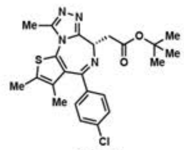

JQ1, 1

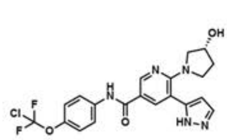

Asciminib, 2

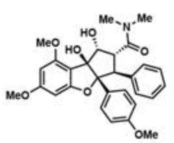

Rocaglamide, 3

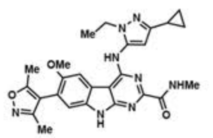

HJB97, 4

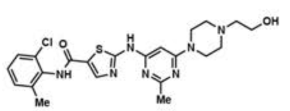

Dasatinib, 5

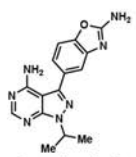

Sapanisertib, 6

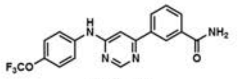

GNF-2, 7

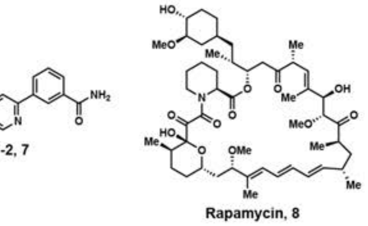

Rapamycin, 8

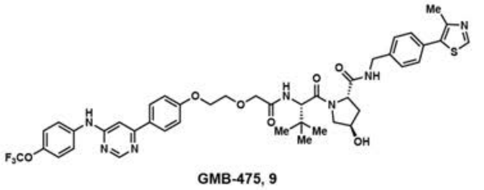

GMB-475, 9

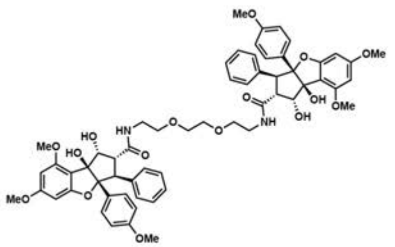

BisRoc-3, 10

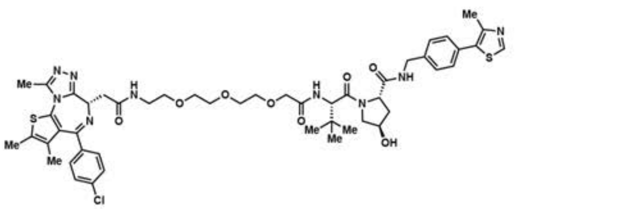

MZ1, 11

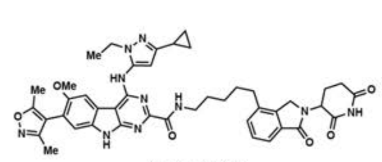

BETd-260, 12

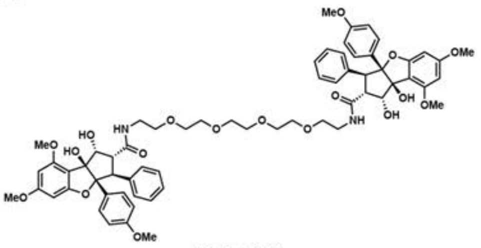

BisRoc-2, 13

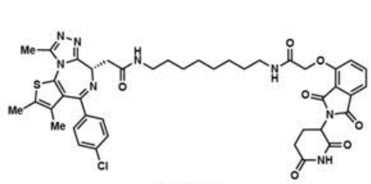

dBET6, 14

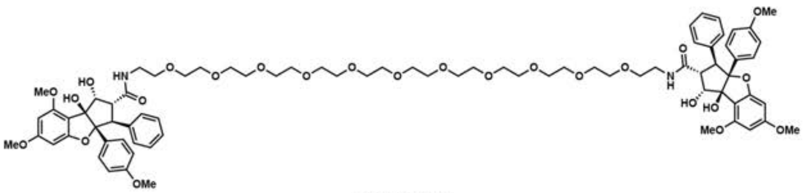

BisRoc-1, 15

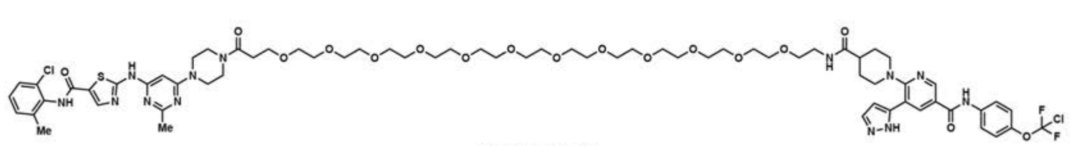

DasatiLink-1, 16

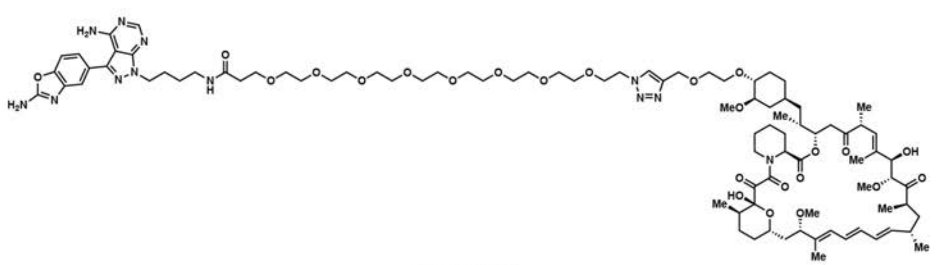

RapaLink-1, 17

Fig. S10. Chemical structures of inhibitors assessed for IFITM assistance.

Table S1. Computed physicochemical properties of compounds described herein.

	MW[a]	Rotatable bonds[b]	cLogP[a]	HBDs[a]	HBAs[a]	TPSA[b]
	≤ 500	≤ 10	≤ 5	≤ 5	≤ 10	≤ 140 Å^2
**Non-linked chemotypes:**						
Asciminib	449.84	7	3.60	3	8	103.37
Dasatinib	488.01	8	3.46	3	9	134.75
GNF-2	374.32	6	4.66	2	6	90.13
HJB97	500.55	8	5.05	3	11	135.78
JQ1	456.99	5	4.97	0	6	97.61
Rapamycin	914.17	6	6.12	3	14	195.43
Rocaglamide	505.56	7	3.05	2	8	97.69
Sapanisertib	309.33	2	3.54	2	8	121.67
TAMRA-N_3	512.56	9	2.94	1	10	138.37
**Linked chemotypes:**						
BETd-260	798.89	15	6.86	4	16	202.26
BisRoc-1	1465.6	50	6.14	6	27	314.49
BisRoc-2	1157.3	29	6.02	6	20	249.88
BisRoc-3	1069.2	23	5.99	6	18	231.42
DasatiLink-1	1517.5	55	8.35	5	30	354.59
dBET6	841.37	18	5.73	3	15	222.29
GMB-475	861.93	21	7.21	4	14	205.37
MZ1	1002.6	25	6.14	4	17	267.97
RapaLink-1	1784.1	46	9.74	5	36	448.98
RapaTAMRA-PEG8	1847.2	44	8.88	3	33	386.83
TAMRA-PEG8-N_3	850.95	32	2.68	1	18	212.21

[a]Guideline based on Lipinski et al. *Adv. Drug Deliver. Rev.* **23**, 3–25 (1997).
[b]Guideline based on Veber et al. *J. Med. Chem*. **45**, 2615–2623 (2002).
MW = molecular weight, cLogP = calculated logarithm of octanol-water partition coefficienct, HBDs = hydrogen bond donors, HBAs = hydrogen bond acceptors, TPSA = topological polar surface area

Table S2. Sequences of sgRNA protospacers described herein.

	**Protospacer sequence**
NegCtrl sg	GAACGACTAGTTAGGCGTGTA
TriNegCtrl sg (1/3)	GACGACTAGTTAGGCGTGTA
TriNegCtrl sg (2/3)	GGCCAAACGTGCCCTGACGG
TriNegCtrl sg (3/3)	GCCTTGGCTAAACCGCTCCC
**CRISPRi:**	
FKBP12 i-sg1	GACGGCTCTGCCTAGTACCT
FKBP12 i-sg2	GCCCAGGAGACGGTGAGTAG
IFITM1 i-sg1	GGTGGAGCGAAGGGCCGCTG
IFITM1 i-sg2	GGAAGGGCCGCTGTGGTGTC
IFITM2 i-sg1	GAGAGAAGGTTTGCACAATG
IFITM2 i-sg2	GTGTGGTTCATGGTGACCAG
IFITM3 i-sg1	GGGTGGAGCTCCAGGCTCAG
IFITM3 i-sg2	GGCACCCTCTGAGCATTCCC
**CRISPRa:**	
FKBP12 a-sg1	GTCCCGGAAACCCAGGCCTC
FKBP12 a-sg2	GGGGCAGGGAGATGCTTAAC
IFITM1 a-sg1	GGGCCCTGGGGATTTTACCC
IFITM1 a-sg2	GGAGGAAAGGCTGAAGGCTA
IFITM2 a-sg1	GAGCTGGCCAGGGCCAGATA
IFITM2 a-sg2	GTCAAATGCAGAGCTGGCCA
IFITM3 a-sg1	GATTTGGCCGGGGCCAGATG
IFITM3 a-sg2	GGAGCCCTGAACCGGGACAG

Data file S1. K562 CRISPRi genome-scale sgRNA counts and phenotypes.

Data file S2. K562 CRISPRi gene phenotypes.

Data file S3. K562 CRISPRa genome-scale sgRNA counts and phenotypes.

Data file S4. K562 CRISPRa gene phenotypes.

Data file S5. Cell line inhibitor sensitivity-gene expression correlation data.

Data file S6. Intracellular kinase occupancy profiling chemoproteomic data.

Data file S7. Cell viability IC_{50} values and fold change calculations for Fig. 4B.

References

1. C. A. Lipinski, F. Lombardo, B. W. Dominy, P. J. Feeney, Experimental and computational approaches to estimate solubility and permeability in drug discovery and development settings. *Adv Drug Deliver Rev*. 23, 3–25 (1997).

2. D. F. Veber, S. R. Johnson, H.-Y. Cheng, B. R. Smith, K. W. Ward, K. D. Kopple, Molecular Properties That Influence the Oral Bioavailability of Drug Candidates. *J Med Chem*. 45, 2615–2623 (2002).

3. A. K. Ghose, V. N. Viswanadhan, J. J. Wendoloski, A Knowledge-Based Approach in Designing Combinatorial or Medicinal Chemistry Libraries for Drug Discovery. 1. A Qualitative and Quantitative Characterization of Known Drug Databases. *J Comb Chem*. 1, 55–68 (1999).

4. M. Erez, A. E. Takemori, P. S. Portoghese, Narcotic antagonistic potency of bivalent ligands which contain .beta.-naltrexamine. Evidence for simultaneous occupation of proximal recognition sites. *J Med Chem*. 25, 847–849 (1982).

5. D. Spencer, T. Wandless, S. Schreiber, G. Crabtree, Controlling signal transduction with synthetic ligands. *Science*. 262, 1019–1024 (1993).

6. K. M. Sakamoto, K. B. Kim, A. Kumagai, F. Mercurio, C. M. Crews, R. J. Deshaies, Protacs: Chimeric molecules that target proteins to the Skp1-Cullin-F box complex for ubiquitination and degradation. *Proc National Acad Sci*. 98, 8554–8559 (2001).

7. V. S. Rodrik-Outmezguine, M. Okaniwa, Z. Yao, C. J. Novotny, C. McWhirter, A. Banaji, H. Won, W. Wong, M. Berger, E. de Stanchina, D. G. Barratt, S. Cosulich, T. Klinowska, N. Rosen, K. M. Shokat, Overcoming mTOR resistance mutations with a new-generation mTOR inhibitor. *Nature*. 534, 272–6 (2016).

8. G. S. Erwin, M. P. Grieshop, A. Ali, J. Qi, M. Lawlor, D. Kumar, I. Ahmad, A. McNally, N. Teider, K. Worringer, R. Sivasankaran, D. N. Syed, A. Eguchi, Md. Ashraf, J. Jeffery, M. Xu, P. M. C. Park, H. Mukhtar, A. K. Srivastava, M. Faruq, J. E. Bradner, A. Z. Ansari, Synthetic transcription elongation factors license transcription across repressive chromatin. *Science*. 358, eaan6414 (2017).

9. M. G. Costales, Y. Matsumoto, S. P. Velagapudi, M. D. Disney, Small Molecule Targeted Recruitment of a Nuclease to RNA. *J Am Chem Soc*. 140, 6741–6744 (2018).

10. S. U. Siriwardena, D. N. P. M. Godage, V. M. Shoba, S. Lai, M. Shi, P. Wu, S. K. Chaudhary, S. L. Schreiber, A. Choudhary, Phosphorylation-Inducing Chimeric Small Molecules. *J Am Chem Soc*. 142, 14052–14057 (2020).

11. N. J. Henning, L. Boike, J. N. Spradlin, C. C. Ward, G. Liu, E. Zhang, B. P. Belcher, S. M. Brittain, M. J. Hesse, D. Dovala, L. M. McGregor, R. V. Misiolek, L. W. Plasschaert, D. J.

Rowlands, F. Wang, A. O. Frank, D. Fuller, A. R. Estes, K. L. Randal, A. Panidapu, J. M. McKenna, J. A. Tallarico, M. Schirle, D. K. Nomura, Deubiquitinase-targeting chimeras for targeted protein stabilization. *Nat Chem Biol*. 18, 412–421 (2022).

12. B. C. Doak, B. Over, F. Giordanetto, J. Kihlberg, Oral Druggable Space beyond the Rule of 5: Insights from Drugs and Clinical Candidates. *Chem Biol*. 21, 1115–1142 (2014).

13. M. Green, P. M. Loewenstein, Autonomous functional domains of chemically synthesized human immunodeficiency virus tat trans-activator protein. *Cell*. 55, 1179–1188 (1988).

14. A. D. Frankel, C. O. Pabo, Cellular uptake of the tat protein from human immunodeficiency virus. *Cell*. 55, 1189–1193 (1988).

15. E. L. Snyder, S. F. Dowdy, Cell Penetrating Peptides in Drug Delivery. *Pharmaceut Res*. 21, 389–393 (2004).

16. M. Békés, D. R. Langley, C. M. Crews, PROTAC targeted protein degraders: the past is prologue. *Nat Rev Drug Discov*. 21, 181–200 (2022).

17. C. A. Foley, F. Potjewyd, K. N. Lamb, L. I. James, S. V. Frye, Assessing the Cell Permeability of Bivalent Chemical Degraders Using the Chloroalkane Penetration Assay. *Acs Chem Biol*. 15, 290–295 (2019).

18. C. Cantrill, P. Chaturvedi, C. Rynn, J. P. Schaffland, I. Walter, M. B. Wittwer, Fundamental aspects of DMPK optimization of targeted protein degraders. *Drug Discov Today*. 25, 969–982 (2020).

19. D. E. Scott, T. P. C. Rooney, E. D. Bayle, T. Mirza, H. M. G. Willems, J. H. Clarke, S. P. Andrews, J. Skidmore, Systematic Investigation of the Permeability of Androgen Receptor PROTACs. *Acs Med Chem Lett* (2020), doi:10.1021/acsmedchemlett.0c00194.

20. G. Ermondi, M. Vallaro, G. Caron, Degraders early developability assessment: face-to-face with molecular properties. *Drug Discov Today*. 25, 1585–1591 (2020).

21. V. G. Klein, C. E. Townsend, A. Testa, M. Zengerle, C. Maniaci, S. J. Hughes, K.-H. Chan, A. Ciulli, R. S. Lokey, Understanding and Improving the Membrane Permeability of VH032-Based PROTACs. *Acs Med Chem Lett*. 11, 1732–1738 (2020).

22. Y. Atilaw, V. Poongavanam, C. S. Nilsson, D. Nguyen, A. Giese, D. Meibom, M. Erdelyi, J. Kihlberg, Solution Conformations Shed Light on PROTAC Cell Permeability. *Acs Med Chem Lett*. 12, 107–114 (2020).

23. A. D. Stasi, S.-K. Tey, G. Dotti, Y. Fujita, A. Kennedy-Nasser, C. Martinez, K. Straathof, E. Liu, A. G. Durett, B. Grilley, H. Liu, C. R. Cruz, B. Savoldo, A. P. Gee, J. Schindler, R. A. Krance, H. E. Heslop, D. M. Spencer, C. M. Rooney, M. K. Brenner, Inducible Apoptosis as a Safety Switch for Adoptive Cell Therapy. *New Engl J Medicine*. 365, 1673–1683 (2011).

24. Q. Fan, O. Aksoy, R. A. Wong, S. Ilkhanizadeh, C. J. Novotny, W. C. Gustafson, A. Y.-Q. Truong, G. Cayanan, E. F. Simonds, D. Haas-Kogan, J. J. Phillips, T. Nicolaides, M. Okaniwa, K. M. Shokat, W. A. Weiss, A Kinase Inhibitor Targeted to mTORC1 Drives Regression in Glioblastoma. *Cancer Cell*. 31, 424–435 (2017).

25. B. J. Lee, J. A. Boyer, G. L. Burnett, A. P. Thottumkara, N. Tibrewal, S. L. Wilson, T. Hsieh, A. Marquez, E. G. Lorenzana, J. W. Evans, L. Hulea, G. Kiss, H. Liu, D. Lee, O. Larsson, S. McLaughlan, I. Topisirovic, Z. Wang, Z. Wang, Y. Zhao, D. Wildes, J. B. Aggen, M. Singh, A. L. Gill, J. A. M. Smith, N. Rosen, Selective inhibitors of mTORC1 activate 4EBP1 and suppress tumor growth. *Nat Chem Biol*. 17, 1065–1074 (2021).

26. H. A. B. III, S. V. Ulahannan, E. B. Haura, S.-H. I. Ou, A. Capasso, P. N. Munster, H. Kitai, Z. Wang, J. Hayes, L. Tao, S. Wong, Y. C. Yang, J. Jiang, B. Bitman, M. Singh, W. C. Gustafson, N. Rosen, A. M. Schram, The bi-steric mTORC1-selective inhibitor RMC-5552 in tumors with activation of mTOR signaling: Preclinical activity in combination with RAS(ON) inhibitors in RAS-addicted tumors, and initial clinical findings from a single agent phase 1/1b study. *J Clin Oncol*. 40, 3098–3098 (2022).

27. Z. Zhang, Q. Fan, X. Luo, K. J. Lou, W. A. Weiss, K. M. Shokat, *Biorxiv*, in press, doi:10.1101/2020.10.12.336677.

28. L. A. Gilbert, M. A. Horlbeck, B. Adamson, J. E. Villalta, Y. Chen, E. H. Whitehead, C. Guimaraes, B. Panning, H. L. Ploegh, M. C. Bassik, L. S. Qi, M. Kampmann, J. S. Weissman, Genome-Scale CRISPR-Mediated Control of Gene Repression and Activation. *Cell*. 159, 647–661 (2014).

29. M. A. Horlbeck, L. A. Gilbert, J. E. Villalta, B. Adamson, R. A. Pak, Y. Chen, A. P. Fields, C. Park, J. E. Corn, M. Kampmann, J. S. Weissman, Compact and highly active next-generation libraries for CRISPR-mediated gene repression and activation. *eLife*. 5, e19760 (2016).

30. M. Jost, Y. Chen, L. A. Gilbert, M. A. Horlbeck, L. Krenning, G. Menchon, A. Rai, M. Y. Cho, J. J. Stern, A. E. Prota, M. Kampmann, A. Akhmanova, M. O. Steinmetz, M. E. Tanenbaum, J. S. Weissman, Combined CRISPRi/a-Based Chemical Genetic Screens Reveal that Rigosertib Is a Microtubule-Destabilizing Agent. *Molecular Cell*. 68, 210-223.e6 (2017).

31. M. Jost, J. S. Weissman, CRISPR Approaches to Small Molecule Target Identification. *Acs Chem Biol*. 13, 366–375 (2018).

32. G. Y. Liu, D. M. Sabatini, mTOR at the nexus of nutrition, growth, ageing and disease. *Nat Rev Mol Cell Bio*. 21, 183–203 (2020).

33. E. D. Zan, R. van Stiphout, B. V. Gapp, V. A. Blomen, T. R. Brummelkamp, S. M. B. Nijman, Quantitative genetic screening reveals a Ragulator-FLCN feedback loop that regulates the mTORC1 pathway. *Sci Signal*. 13, eaba5665 (2020).

34. K. J. Condon, J. M. Orozco, C. H. Adelmann, J. B. Spinelli, P. W. van der Helm, J. M. Roberts, T. Kunchok, D. M. Sabatini, Genome-wide CRISPR screens reveal multitiered mechanisms through which mTORC1 senses mitochondrial dysfunction. *Proc National Acad Sci*. 118, e2022120118 (2021).

35. Z. Zhang, J. Liu, M. Li, H. Yang, C. Zhang, Evolutionary Dynamics of the Interferon-Induced Transmembrane Gene Family in Vertebrates. *Plos One*. 7, e49265 (2012).

36. P. Li, M.-L. Shi, W.-L. Shen, Z. Zhang, D.-J. Xie, X.-Y. Zhang, C. He, Y. Zhang, Z.-H. Zhao, Coordinated regulation of IFITM1, 2 and 3 genes by an IFN-responsive enhancer through long-range chromatin interactions. *Biochimica Et Biophysica Acta Bba - Gene Regul Mech*. 1860, 885–893 (2017).

37. M. J. Garnett, E. J. Edelman, S. J. Heidorn, C. D. Greenman, A. Dastur, K. W. Lau, P. Greninger, I. R. Thompson, X. Luo, J. Soares, Q. Liu, F. Iorio, D. Surdez, L. Chen, R. J. Milano, G. R. Bignell, A. T. Tam, H. Davies, J. A. Stevenson, S. Barthorpe, S. R. Lutz, F. Kogera, K. Lawrence, A. McLaren-Douglas, X. Mitropoulos, T. Mironenko, H. Thi, L. Richardson, W. Zhou, F. Jewitt, T. Zhang, P. O'Brien, J. L. Boisvert, S. Price, W. Hur, W. Yang, X. Deng, A. Butler, H. G. Choi, J. W. Chang, J. Baselga, I. Stamenkovic, J. A. Engelman, S. V. Sharma, O. Delattre, J. Saez-Rodriguez, N. S. Gray, J. Settleman, P. A. Futreal, D. A. Haber, M. R. Stratton, S. Ramaswamy, U. McDermott, C. H. Benes, Systematic identification of genomic markers of drug sensitivity in cancer cells. *Nature*. 483, 570–575 (2012).

38. M. G. Rees, B. Seashore-Ludlow, J. H. Cheah, D. J. Adams, E. V. Price, S. Gill, S. Javaid, M. E. Coletti, V. L. Jones, N. E. Bodycombe, C. K. Soule, B. Alexander, A. Li, P. Montgomery, J. D. Kotz, C. S.-Y. Hon, B. Munoz, T. Liefeld, V. Dančík, D. A. Haber, C. B. Clish, J. A. Bittker, M. Palmer, B. K. Wagner, P. A. Clemons, A. F. Shamji, S. L. Schreiber, Correlating chemical sensitivity and basal gene expression reveals mechanism of action. *Nat Chem Biol*. 12, 109–116 (2016).

39. F. Iorio, T. A. Knijnenburg, D. J. Vis, G. R. Bignell, M. P. Menden, M. Schubert, N. Aben, E. Gonçalves, S. Barthorpe, H. Lightfoot, T. Cokelaer, P. Greninger, E. van Dyk, H. Chang, H. de Silva, H. Heyn, X. Deng, R. K. Egan, Q. Liu, T. Mironenko, X. Mitropoulos, L. Richardson, J. Wang, T. Zhang, S. Moran, S. Sayols, M. Soleimani, D. Tamborero, N. Lopez-Bigas, P. Ross-Macdonald, M. Esteller, N. S. Gray, D. A. Haber, M. R. Stratton, C. H. Benes, L. F. A. Wessels, J. Saez-Rodriguez, U. McDermott, M. J. Garnett, A Landscape of Pharmacogenomic Interactions in Cancer. *Cell*. 166, 740–754 (2016).

40. B. Adamson, T. M. Norman, M. Jost, M. Y. Cho, J. K. Nuñez, Y. Chen, J. E. Villalta, L. A. Gilbert, M. A. Horlbeck, M. Y. Hein, R. A. Pak, A. N. Gray, C. A. Gross, A. Dixit, O. Parnas, A. Regev, J. S. Weissman, A Multiplexed Single-Cell CRISPR Screening Platform Enables Systematic Dissection of the Unfolded Protein Response. *Cell*. 167, 1867-1882.e21 (2016).

41. B. R. Stockwell, Chemical genetics: ligand-based discovery of gene function. *Nat Rev Genet*. 1, 116–125 (2000).

42. A. L. Brass, I.-C. Huang, Y. Benita, S. P. John, M. N. Krishnan, E. M. Feeley, B. J. Ryan, J. L. Weyer, L. van der Weyden, E. Fikrig, D. J. Adams, R. J. Xavier, M. Farzan, S. J. Elledge, The IFITM Proteins Mediate Cellular Resistance to Influenza A H1N1 Virus, West Nile Virus, and Dengue Virus. *Cell*. 139, 1243–1254 (2009).

43. C. C. Bailey, G. Zhong, I.-C. Huang, M. Farzan, IFITM-Family Proteins: The Cell's First Line of Antiviral Defense. *Annual Review of Virology*. 1, 1–23 (2014).

44. G. Shi, O. Schwartz, A. A. Compton, More than meets the I: the diverse antiviral and cellular functions of interferon-induced transmembrane proteins. *Retrovirology*. 14, 53 (2017).

45. X. Zhao, J. Li, C. A. Winkler, P. An, J.-T. Guo, IFITM Genes, Variants, and Their Roles in the Control and Pathogenesis of Viral Infections. *Frontiers in Microbiology*. 9, 3228 (2019).

46. K. Li, R. M. Markosyan, Y.-M. Zheng, O. Golfetto, B. Bungart, M. Li, S. Ding, Y. He, C. Liang, J. C. Lee, E. Gratton, F. S. Cohen, S.-L. Liu, IFITM Proteins Restrict Viral Membrane Hemifusion. *Plos Pathog*. 9, e1003124 (2013).

47. X. Zhao, F. Guo, F. Liu, A. Cuconati, J. Chang, T. M. Block, J.-T. Guo, Interferon induction of IFITM proteins promotes infection by human coronavirus OC43. *Proc National Acad Sci*. 111, 6756–6761 (2014).

48. J. Lee, M. E. Robinson, N. Ma, D. Artadji, M. A. Ahmed, G. Xiao, T. Sadras, G. Deb, J. Winchester, K. N. Cosgun, H. Geng, L. N. Chan, K. Kume, T. P. Miettinen, Y. Zhang, M. A. Nix, L. Klemm, C. W. Chen, J. Chen, V. Khairnar, A. P. Wiita, A. Thomas-Tikhonenko, M. Farzan, J. U. Jung, D. M. Weinstock, S. R. Manalis, M. S. Diamond, N. Vaidehi, M. Müschen, IFITM3 functions as a PIP3 scaffold to amplify PI3K signalling in B cells. *Nature*, 1–7 (2020).

49. J. Buchrieser, S. A. Degrelle, T. Couderc, Q. Nevers, O. Disson, C. Manet, D. A. Donahue, F. Porrot, K.-H. Hillion, E. Perthame, M. V. Arroyo, S. Souquere, K. Ruigrok, A. Dupressoir, T. Heidmann, X. Montagutelli, T. Fournier, M. Lecuit, O. Schwartz, IFITM proteins inhibit placental syncytiotrophoblast formation and promote fetal demise. *Sci New York N Y*. 365, 176–180 (2019).

50. J. S. Spence, R. He, H.-H. Hoffmann, T. Das, E. Thinon, C. M. Rice, T. Peng, K. Chandran, H. C. Hang, IFITM3 directly engages and shuttles incoming virus particles to lysosomes. *Nat Chem Biol*. 15, 259–268 (2019).

51. T. Das, X. Yang, H. Lee, E. H. Garst, E. Valencia, K. Chandran, W. Im, H. C. Hang, S-Palmitoylation and Sterol Interactions Mediate Antiviral Specificity of IFITMs. *Acs Chem Biol*. 17, 2109–2120 (2022).

52. F. H. Epstein, R. Kurzrock, J. U. Gutterman, M. Talpaz, The Molecular Genetics of Philadelphia Chromosome–Positive Leukemias. *New Engl J Medicine*. 319, 990–998 (1988).

53. T. Schindler, W. Bornmann, P. Pellicena, W. T. Miller, B. Clarkson, J. Kuriyan, Structural Mechanism for STI-571 Inhibition of Abelson Tyrosine Kinase. *Science*. 289, 1938–1942 (2000).

54. T. P. Braun, C. A. Eide, B. J. Druker, Response and Resistance to BCR-ABL1-Targeted Therapies. *Cancer Cell*. 37, 530–542 (2020).

55. J. Zhang, F. J. Adrián, W. Jahnke, S. W. Cowan-Jacob, A. G. Li, R. E. Iacob, T. Sim, J. Powers, C. Dierks, F. Sun, G.-R. Guo, Q. Ding, B. Okram, Y. Choi, A. Wojciechowski, X. Deng, G. Liu, G. Fendrich, A. Strauss, N. Vajpai, S. Grzesiek, T. Tuntland, Y. Liu, B. Bursulaya, M. Azam, P. W. Manley, J. R. Engen, G. Q. Daley, M. Warmuth, N. S. Gray, Targeting Bcr–Abl by combining allosteric with ATP-binding-site inhibitors. *Nature*. 463, 501–506 (2010).

56. A. A. Wylie, J. Schoepfer, W. Jahnke, S. W. Cowan-Jacob, A. Loo, P. Furet, A. L. Marzinzik, X. Pelle, J. Donovan, W. Zhu, S. Buonamici, A. Q. Hassan, F. Lombardo, V. Iyer, M. Palmer, G. Berellini, S. Dodd, S. Thohan, H. Bitter, S. Branford, D. M. Ross, T. P. Hughes, L. Petruzzelli, K. G. Vanasse, M. Warmuth, F. Hofmann, N. J. Keen, W. R. Sellers, The allosteric inhibitor ABL001 enables dual targeting of BCR–ABL1. *Nature*. 543, 733–737 (2017).

57. R. E. Iacob, J. Zhang, N. S. Gray, J. R. Engen, Allosteric Interactions between the Myristate- and ATP-Site of the Abl Kinase. *Plos One*. 6, e15929 (2011).

58. N. Vajpai, A. Strauss, G. Fendrich, S. W. Cowan-Jacob, P. W. Manley, S. Grzesiek, W. Jahnke, Solution Conformations and Dynamics of ABL Kinase-Inhibitor Complexes Determined by NMR Substantiate the Different Binding Modes of Imatinib/Nilotinib and Dasatinib* ♦. *J Biol Chem*. 283, 18292–18302 (2008).

59. M. A. Fabian, W. H. Biggs, D. K. Treiber, C. E. Atteridge, M. D. Azimioara, M. G. Benedetti, T. A. Carter, P. Ciceri, P. T. Edeen, M. Floyd, J. M. Ford, M. Galvin, J. L. Gerlach, R. M. Grotzfeld, S. Herrgard, D. E. Insko, M. A. Insko, A. G. Lai, J.-M. Lélias, S. A. Mehta, Z. V. Milanov, A. M. Velasco, L. M. Wodicka, H. K. Patel, P. P. Zarrinkar, D. J. Lockhart, A small molecule–kinase interaction map for clinical kinase inhibitors. *Nat Biotechnol*. 23, 329–336 (2005).

60. J. Das, P. Chen, D. Norris, R. Padmanabha, J. Lin, R. V. Moquin, Z. Shen, L. S. Cook, A. M. Doweyko, S. Pitt, S. Pang, D. R. Shen, Q. Fang, H. F. de Fex, K. W. McIntyre, D. J. Shuster, K. M. Gillooly, K. Behnia, G. L. Schieven, J. Wityak, J. C. Barrish, 2-Aminothiazole as a Novel Kinase Inhibitor Template. Structure−Activity Relationship Studies toward the Discovery of N -(2-Chloro-6-methylphenyl)-2-[[6-[4-(2-hydroxyethyl)-1- piperazinyl)]-2-methyl-4-pyrimidinyl]amino)]-1,3-thiazole-5-carboxamide (Dasatinib, BMS-354825) as a Potent pan -Src Kinase Inhibitor. *J Med Chem*. 49, 6819–6832 (2006).

61. C. M. Gower, M. E. K. Chang, D. J. Maly, Bivalent inhibitors of protein kinases. *Crit Rev Biochem Mol*. 49, 102–115 (2014).

62. Q. Zhao, X. Ouyang, X. Wan, K. S. Gajiwala, J. C. Kath, L. H. Jones, A. L. Burlingame, J. Taunton, Broad-Spectrum Kinase Profiling in Live Cells with Lysine-Targeted Sulfonyl Fluoride Probes. *Journal of the American Chemical Society*. 139, 680–685 (2017).

63. B. F. Cravatt, A. T. Wright, J. W. Kozarich, Activity-Based Protein Profiling: From Enzyme Chemistry to Proteomic Chemistry. *Annu Rev Biochem*. 77, 383–414 (2008).

64. S. Iwasaki, S. N. Floor, N. T. Ingolia, Rocaglates convert DEAD-box protein eIF4A into a sequence-selective translational repressor. *Nature*. 534, 558 (2016).

65. S. Iwasaki, W. Iwasaki, M. Takahashi, A. Sakamoto, C. Watanabe, Y. Shichino, S. N. Floor, K. Fujiwara, M. Mito, K. Dodo, M. Sodeoka, H. Imataka, T. Honma, K. Fukuzawa, T. Ito, N. T. Ingolia, The Translation Inhibitor Rocaglamide Targets a Bimolecular Cavity between eIF4A and Polypurine RNA. *Mol Cell*. 73, 738-748.e9 (2019).

66. C. M. Dobson, Chemical space and biology. *Nature*. 432, 824–828 (2004).

67. D. R. Wassarman, dwassarman/cellpanelr: v0.0.0.9001, Zenodo (2022); 10.5281/zenodo.7191410.

68. M. A. Horlbeck, X. Xiong, M. Jost, mhorlbeck/ScreenProcessing: v0.1, Zenodo (2022); 10.5281/zenodo.7185885.

69. S. J. Liu, M. A. Horlbeck, J. S. Weissman, D. A. Lim, Functional Analysis of Long Non-Coding RNAs, Methods and Protocols. *Methods Mol Biology*. 2254, 323–338 (2020).

70. M. Ghandi, F. W. Huang, J. Jané-Valbuena, G. V. Kryukov, C. C. Lo, E. R. McDonald, J. Barretina, E. T. Gelfand, C. M. Bielski, H. Li, K. Hu, A. Y. Andreev-Drakhlin, J. Kim, J. M. Hess, B. J. Haas, F. Aguet, B. A. Weir, M. V. Rothberg, B. R. Paolella, M. S. Lawrence, R. Akbani, Y. Lu, H. L. Tiv, P. C. Gokhale, A. de Weck, A. A. Mansour, C. Oh, J. Shih, K. Hadi, Y. Rosen, J. Bistline, K. Venkatesan, A. Reddy, D. Sonkin, M. Liu, J. Lehar, J. M. Korn, D. A. Porter, M. D. Jones, J. Golji, G. Caponigro, J. E. Taylor, C. M. Dunning, A. L. Creech, A. C. Warren, J. M. McFarland, M. Zamanighomi, A. Kauffmann, N. Stransky, M. Imielinski, Y. E. Maruvka, A. D. Cherniack, A. Tsherniak, F. Vazquez, J. D. Jaffe, A. A. Lane, D. M. Weinstock, C. M. Johannessen, M. P. Morrissey, F. Stegmeier, R. Schlegel, W. C. Hahn, G. Getz, G. B. Mills, J. S. Boehm, T. R. Golub, L. A. Garraway, W. R. Sellers, Next-generation characterization of the Cancer Cell Line Encyclopedia. *Nature*. 569, 503–508 (2019).

71. H. Wickham, M. Averick, J. Bryan, W. Chang, L. McGowan, R. François, G. Grolemund, A. Hayes, L. Henry, J. Hester, M. Kuhn, T. Pedersen, E. Miller, S. Bache, K. Müller, J. Ooms, D. Robinson, D. Seidel, V. Spinu, K. Takahashi, D. Vaughan, C. Wilke, K. Woo, H. Yutani, Welcome to the Tidyverse. *J Open Source Softw*. 4, 1686 (2019).

72. M. A. Seeliger, M. Young, M. N. Henderson, P. Pellicena, D. S. King, A. M. Falick, J. Kuriyan, High yield bacterial expression of active c-Abl and c-Src tyrosine kinases. *Protein Sci*. 14, 3135–3139 (2005).

73. E. Gasteiger, C. Hoogland, A. Gattiker, S. Duvaud, M. R. Wilkins, R. D. Appel, A. Bairoch, The Proteomics Protocols Handbook, 571–607 (2005).

74. N. Vajpai, A. Strauss, G. Fendrich, S. W. Cowan-Jacob, P. W. Manley, W. Jahnke, S. Grzesiek, Backbone NMR resonance assignment of the Abelson kinase domain in complex with imatinib. *Biomol Nmr Assigm*. 2, 41–42 (2008).

75. F. Carles, S. Bourg, C. Meyer, P. Bonnet, PKIDB: A Curated, Annotated and Updated Database of Protein Kinase Inhibitors in Clinical Trials. *Molecules*. 23, 908 (2018).

76. G. Weng, C. Shen, D. Cao, J. Gao, X. Dong, Q. He, B. Yang, D. Li, J. Wu, T. Hou, PROTAC-DB: an online database of PROTACs. *Nucleic Acids Res*. 49, gkaa807- (2020).

77. R. Kiss, M. Sandor, F. A. Szalai, http://Mcule.com: a public web service for drug discovery. *J Cheminformatics*. 4, P17 (2012).
